# The Latent Structure of Negative Symptoms in the General Population in Adolescence and Emerging Adulthood

**DOI:** 10.1093/schizbullopen/sgac009

**Published:** 2022-01-12

**Authors:** Laura Havers, Alastair Cardno, Daniel Freeman, Angelica Ronald

**Affiliations:** 1 Department of Psychological Sciences, Birkbeck, University of London, London, UK; 2 Division of Psychological and Social Medicine, University of Leeds, Leeds, UK; 3 Department of Psychiatry, University of Oxford, Oxford, UK; 4 Oxford Health NHS Foundation Trust, Oxford, UK

**Keywords:** psychosis continuum, confirmatory factor analysis, measurement invariance, polygenic scores, subdomain-specificity

## Abstract

Negative symptoms predict adverse outcomes within psychotic disorders, in individuals at high-risk for psychosis, and in young people in the community. There is considerable interest in the dimensional structure of negative symptoms in clinical samples, and accumulating evidence suggests a 5-factor structure. Little is known about the underlying structure of negative symptoms in young people despite the importance of this developmental stage for mental health. We used confirmatory factor analysis to test the structure of parent-reported negative symptoms at mean ages 16.32 (SD 0.68, *N* = 4974), 17.06 (SD 0.88, *N* = 1469) and 22.30 (SD 0.93, *N* = 5179) in a community sample. Given previously reported associations between total negative symptoms and genome-wide polygenic scores (GPS) for major depressive disorder (MDD) and schizophrenia in adolescence, we assessed associations between individual subdomains and these GPSs. A 5-factor model of flat affect, alogia, avolition, anhedonia, and asociality provided the best fit at each age and was invariant over time. The results of our linear regression analyses showed associations between MDD GPS with avolition, flat affect, anhedonia, and asociality, and between schizophrenia GPS with avolition and flat affect. We showed that a 5-factor structure of negative symptoms is present from ages 16 to 22 in the community. Avolition was most consistently associated with polygenic liability to MDD and schizophrenia, and alogia was least associated. These findings highlight the value of dissecting negative symptoms into psychometrically derived subdomains and may offer insights into early manifestation of genetic risk for MDD and schizophrenia.

## Introduction

It is well documented that negative symptoms in schizophrenia are associated with a range of poor functional and clinical outcomes.^1–4^ They are also associated with transition to psychotic disorder in individuals at clinical high risk for psychosis^5–7^ and are often the first signs of emergent schizophrenia.^8^ Despite collective evidence suggesting the importance of these “early” negative symptoms,^9^ they remain poorly understood.^10^ Furthermore, the putative psychosis prodrome is characterized primarily in terms of positive symptoms^10,11^ (e.g., delusions, hallucinations) and characterization of negative symptoms is currently limited (though see^12–14^). Negative symptoms, which include deficits in emotional expressivity (flat affect), speech production (alogia), goal-oriented motivation (avolition), derivation of pleasure (anhedonia), and social engagement (asociality), are also seen in attenuated form in the general population.^15,16^

Adolescence is a key developmental stage for the onset of mental health problems,^17^ yet there is a paucity of research into negative symptoms reported by young people in the community. Presence of these symptoms also appears to represent a marker for suboptimal course,^18^ particularly when they co-occur with positive psychotic experiences.^19,20^ In a study of 14–24 year-olds, negative symptoms (together with an item about disorganization) predicted incidence and persistence of positive symptoms, and co-occurrence of the symptoms more strongly predicted psychotic impairment compared to positive symptoms alone.^19^ In a quasi-longitudinal study of 25–34 year-olds, negative symptoms in the presence of frequent psychotic experiences predicted transition to schizophrenia.^20^ Identifying aspects of continuity or discontinuity between these, prodromal, and clinical negative symptoms may contribute to unraveling the mechanisms involved in their development.

This can be considered important in the context that there is currently no consensus on effective treatment for idiopathic negative symptoms in schizophrenia.^21–23^ Whilst findings suggest some efficacy of psychosocial and pharmacological treatments,^24,25^ several methodological issues limit the conclusiveness of the findings. Recent evidence supporting a reconceptualization of the negative symptoms construct in terms of its latent structure could prove an important catalyst for improving understanding and treatment.^26^

In the current Diagnostic and Statistical Manual (DSM-5),^27^ negative symptoms in schizophrenia are encapsulated by 2 dimensions reflecting deficits in motivation and pleasure, and in emotional expression.^28^ It has been discussed that this bifurcated conceptualization was founded on exploratory factor analysis (EFA) research that identified 2 factors.^29,30^ However, EFA does not assess factor structure relative to other hypothesized models,^31,32^ and collective evidence that has compared models using confirmatory factor analysis (CFA) as well as network analysis^33^ has cast doubt over the validity of the 2-factor conceptualization (though see^34^). Recent findings from across rating scales,^29^ different cultures^30,35^ and different stages of illness^36^ converge to suggest that the 5 subdomains of blunted affect, alogia, avolition, anhedonia, and asociality, as advanced by the National Institute of Mental Health (NIMH) consensus conference, best describe the negative symptoms construct.^26^ There is also empirical support for hierarchical models that have specified these 5 factors with motivation-pleasure and emotional expression as second-order factors, although the 5-factor model has outperformed hierarchical models across studies^29,30,36^ with 1 exception.^37^

In the community, a single study to date has used CFA to investigate the latent structure of negative symptoms independently of other symptoms.^38^ This study reported a hierarchical structure of self-reported negative symptoms measured in individuals aged 11–18, including the 5 NIMH dimensions plus a second-order factor reflecting total negative symptoms. The study did not test a 2-factor model directly reflecting the DSM conceptualization nor include these 2 factors in a hierarchical model. Other studies of negative symptoms independent of other symptoms are limited to exploratory methods using EFA. These studies identified a 3-factor structure of the Community Assessment of Psychic Experiences (CAPE^39^) self-reported negative subscale in adolescence,^16^ and in individuals aged 12–35.^40^ Three factors of flat affect, avolition, and social withdrawal were identified; however, competing theory-based models with a greater (or fewer) number of factors were not tested. A recent CFA of schizotypal personality traits (a related construct) in adolescence using the Schizotypal Personality Questionnaire for Children (SPQ-C^41^) found a 3-factor structure of negative (interpersonal), disorganized, and positive (cognitive-perceptual) domains,^42^ similar to that found in adults,^43^ though negative/ interpersonal schizotypy has not been analyzed as a separate dimension.

Until recently, endeavors to understand negative symptoms on a mechanistic level and to develop treatment targets have largely been predicated on either a unidimensional or 2-dimensional conceptualization^24,44^: Whilst the 2 dimensions of motivation-pleasure and emotional expression appear to show some correspondence to current understandings of the neurobiology of negative symptoms,^45^ more research is needed in order to identify and probe correlates of the 5 individual subdomains.^27,46,47^ Some specificity has been reported in neural response patterns^48^ and neuropsychological processes across the subdomains,^47^ though at present, etiological mechanisms remain unclear.^46^

It is noted that 2 studies suggest the influence of genetic (familial) risk for schizophrenia on aberrant reward processing, which may be related to the motivational impairments within negative symptoms.^49–51^ At a broader level, there is preliminary evidence for associations between several specific genetic variants and total negative symptoms in schizophrenia,^46,52^ and between polygenic liability to schizophrenia and these symptoms^53–55^ (though see^56^). In the community, associations have also been reported between total negative symptoms in adolescence and polygenic liability to major depressive disorder (MDD)^57^ as well as schizophrenia.^57,58^ Nonetheless, it is not known what symptom-level dimensions drive these associations.

The first aim of the current study was to establish the underlying structure of observer-rated negative symptoms in the community in adolescence and emerging adulthood. CFA of 4 theory-based models (1-factor, 2-factor, 5-factor, 5-factor hierarchical) in-line with those tested previously^29^ and a model derived through EFA was carried out in a longitudinal cohort at 3 ages. It was hypothesized that 5 factors would provide the best representation of the data at each age and that the structure would be invariant across age. The second aim was to investigate whether the identified subdomains were associated with GPS for schizophrenia and MDD. It was hypothesized that the most consistent subdomain-specific associations would be observed for avolition in light of findings suggesting that avolition may be a particularly central symptom within the negative symptoms construct.^59–61^

## Methods

### Participants

Participants were part of the Twins Early Development Study (TEDS). Parents of all twins born between 1994–1996 in England and Wales were invited to take part.^62^ Sixteen thousand eight hundred and ten (16 810) families responded to this initial study invitation.^63^Supplementary tables 1–2 show details of subsequent study participation and exclusions. Parents completed assessments of their twins’ negative symptoms at mean ages 16.32 (SD 0.68, *N* = 4974), 17.06 (SD 0.88, *N* = 1469) and 22.30 (SD 0.93, *N* = 5179). Twin analyses were not conducted in the current study because twin analyses were not part of our aims (see *Statistical Analyses*).

### Negative Symptoms

Negative symptoms were assessed using the 10-item subscale of the Specific Psychotic Experiences Questionnaire (SPEQ).^15^ The SPEQ is a multi-dimensional measure of positive psychotic experiences and negative symptoms in the community, with 6 components (self-reported paranoia, hallucinations, cognitive disorganization, grandiosity, and anhedonia, and parent-reported negative symptoms) identified through principal components analysis.^15^ The negative symptoms subscale was based on the Scale for the Assessment of Negative Symptoms (SANS),^64^ adapted for an adolescent community sample and judged in terms of content validity by clinical collaborators (DF and AC, see^15^). Parents were asked to rate how strongly they agreed or disagreed (“not at all”, “somewhat true”, “mainly true”, “definitely true”) with the items (Supplementary information 1). Two items relating to attentional deficits were excluded in-line with current conceptualizations of negative symptoms.^26,34^ The included items showed good internal consistency (α = 0.83–0.88). Descriptive statistics are reported in Supplementary table 2.

### Genome-Wide Polygenic Scores

Genotyping of participants^65^ is described in Supplementary information 2. Approximately 60% of the phenotypic sample at each age were genotyped (60.31% at 16, 60.91% at 17, 60.19% at 22). Genome-wide polygenic scores (GPS)^66^ were calculated^67^ using LDpred software,^68^ described in Supplementary information 3.^69,70^ GPS were regressed on the first 10 principal components (PCs) of ancestry, batch, and chip. Standardized residuals were used in the analyses. The 1^st^ and 2^nd^ PCs are plotted in Supplementary figure 1. GPS available to TEDS collaborators are based on 3 different fractions (*f*) of causal markers (1, 0.3, 0.01), utilized here to identify the most predictive fractions (Supplementary information 4). GPS decile plots are shown in Supplementary figures 2–3.

### Statistical Analyses

CFA was used to assess the latent structure of negative symptoms. At each age, 4 theory-based models and a model derived through EFA were tested (Supplementary information 5). By virtue of using data from twins, we utilized the 2 phenotypic “subsamples” to run the models in the “main” sample (comprised of one randomly selected individual per pair) and in the co-twin sample, as a pseudoreplication. Absolute fit of the models was assessed using the comparative fit index (CFI), the root mean square error of approximation (RMSEA) and the standardized root mean square residual (SRMR, Supplementary information 6). CFI values >0.95, RMSEA values <0.06 and SRMR values <0.08 indicated acceptable fit.^71^ Relative fit was assessed using the Bayesian Information Criterion (BIC), with lower values indicating better fit. Difference values >2 suggest positive evidence and difference values >10 suggest very strong evidence.^72^ Akaike’s Information Criterion (AIC) was referred to where BIC difference values were <2, with lower values indicating better fit and difference values >2 suggesting strong evidence.^73^

Longitudinal measurement invariance of the factor structure between ages was tested in the subsamples separately (Supplementary information 7). Acceptable fit of each model (configural, metric, scalar, strict) was required to test sequential models. Negligible change in fit between models was required in order to conclude the level of invariance, specifically, CFI < 0.01, RMSEA < 0.015, and SRMR < 0.03.^74^ Measurement invariance of the factor structure was assessed between the main and co-twin samples, with adjusted SE to account for nonindependence of the data.

Parent response rates and demographics are shown in Supplementary table 3. Proportions of item-level data present are shown in Supplementary table 4. Data was assumed missing at random. Full information maximum likelihood (FIML) estimation with Huber-White robust SE and Yuan Bentler adjusted test statistic to correct for multivariate non-normality of the indicators was used (MLR). Data were modeled as continuous. In response to reviewer comments, data were also modeled as categorical using diagonally weighted least squares estimation with robust SE (WLSMV). Cross-sectional model-fitting was conducted using lavaan^75^ in R (version 3.6.2). Longitudinal measurement invariance and categorical models were run in Mplus (version 8.4).

GPS associations with negative symptoms were tested using linear regressions using data from both unrelated individuals (~60%) and related individuals (~40%) with adjusted SE, using MLR in lavaan. Subdomain mean scores at each age were first regressed separately on MDD and schizophrenia GPS at each *f*. The False Discovery Rate (FDR)^76^ method was used to correct for multiple testing at *q* <.05 (Supplementary information 4). Equality of the standardized regression coefficients was tested using the lavTestWald function.^77^ For each subdomain at each age, the most predictive *f* were used in multiple regressions with MDD and schizophrenia GPS as joint predictors.

## Results

### CFA

The 5-factor model had the best standalone fit at all ages in the main sample (CFI >= 0.99, RMSEA <= 0.06, SRMR <= 0.02; table 1). At 16 and 22, BIC values were lower to a magnitude >100 for the 5-factor model compared to the next best fitting models (4-factor EFA and 5-factor hierarchical). At 17, the 5-factor model slightly outperformed the 4-factor EFA model in terms of RMSEA and SRMR, though CFI and BIC values were indistinguishable. The difference in AIC (>2) indicated better fit of the 5-factor model. A 5-factor model also fit the data best at all ages in the co-twin sample (Supplementary table 5). Strict measurement invariance of the 5-factor structure between the subsamples at each age was found (Supplementary tables 6–8). Superior fit of the 5-factor model at each age was also found for the categorical models (Supplementary table 9).

**Table 1.** Confirmatory Factor Analysis of Negative Symptoms: Model Fit Results

	Parameters	Log-likelihood	AIC	BIC	χ ^2^ value (*df*)	CFI	RMSEA [90% CI]	SRMR
*16 years*								
1-factor model	24	–28 955.59	57 959.18	58 115.47	1378.97 (20), *P <* .001	0.78	0.18 [0.17, 0.19]	0.08
2-factor model	25	–27 993.87	56 037.73	56 200.53	547.37 (19), *P <* .001	0.91	0.12 [0.11, 0.12]	0.06
4-factor model	30	–27 479.64	55 019.27	55 214.63	115.81 (14), *P <* .001	0.98	0.06 [0.05, 0.07]	0.03
**5-factor model**	**32**	**–27 382.81**	**54 829.63**	**55 038.01**	**31.48 (12), *P <* .001**	**0.99**	**0.03 [0.02, 0.04]**	**0.01**
5H-factor model	28	–27 509.06	55 074.12	55 256.46	139.67 (16), *P <* .001	0.98	0.06 [0.05, 0.07]	0.03
*17 years*								
1-factor model	24	–9753.86	19 555.72	19 682.73	444.50 (20), *P* < .001	0.85	0.17 [0.15, 0.18]	0.06
2-factor model	25	–9463.35	18 976.70	19 109.01	148.52 (19), *P* < .001	0.95	0.10 [0.08, 0.11]	0.04
4-factor model	30	–9333.55	18 727.11	18 885.88	16.75 (14), *P* = .27	1.00	0.02 [0.00, 0.04]	0.02
**5-factor model**	**32**	**–9325.68**	**18 715.35**	**18 884.71**	**8.40 (12), *P* = .75**	**1.00**	**0.00 [0.00, 0.03]**	**0.01**
5H-factor model	28	–9336.36	18 728.73	18 876.91	19.59 (16), *P* = .24	1.00	0.02 [0.00, 0.04]	0.02
*22 years*								
1-factor model	24	–34 446.79	68 941.58	69 098.84	940.15 (20), *P* < .001	0.86	0.14 [0.13, 0.15]	0.06
2-factor model	25	–33 945.17	67 940.34	68 104.15	480.38 (19), *P* < .001	0.93	0.10 [0.09, 0.11]	0.05
4-factor model	29	–33 658.92	67 375.84	67 565.86	217.03 (15), *P* < .001	0.97	0.07 [0.06, 0.08]	0.03
**5-factor model**	**32**	**–33 554.72**	**67 173.44**	**67 383.11**	**110.13 (12), *P* < .001**	**0.99**	**0.06 [0.05, 0.07]**	**0.02**
5H-factor model	28	–33 633.77	67 323.54	67 507.01	185.89 (16), *P* < .001	0.98	0.07 [0.06, 0.07]	0.03

*Note: N* age 16 *=* 4974; *N* age 17 *=* 1469; *N* age 22 *=* 5179. Robust maximum likelihood estimation (MLR). 5H-factor model, 5-factor hierarchical model; AIC, Akaike’s Information Criterion; BIC, Bayesian Information Criterion; χ ^2^, chi-square value; CFI, comparative fit index; RMSEA, root mean square error of approximation; SRMR, standardized root mean square residual. Baseline models: At 16, χ ^2^ (28) = 5626.51, *P <* .001. At 17, χ ^2^ (28) = 2643.21, *P <* .001. At 22, χ ^2^ (28) = 6163.17, *P <* .001. Bold typeset represents best fitting model at each age.

### Latent Factors

**Figure 1.**
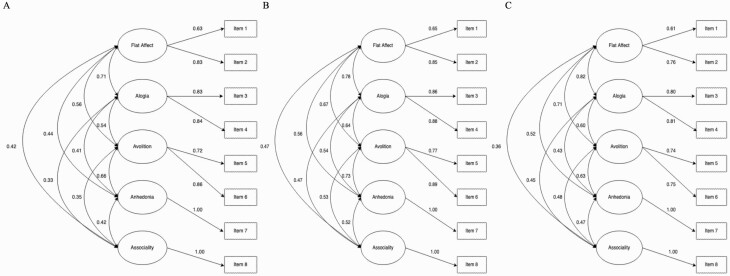
Five-factor model of negative symptoms at ages 16, 17, and 22. (A) Age 16; (B) Age 17; (C) Age 22. Standardized estimates from best fitting confirmatory factor analysis models. Rectangles represent measured variables. Circles represent latent variables. Double-headed arrows represent correlations. Single-headed arrows represent factor loadings.

Parameter estimates are reported from the 5-factor model at each age in Supplementary tables 10–12. Latent factors were defined as flat affect, alogia, avolition, anhedonia, and asociality. Correlations between latent factors were moderate to high (.33 to .82). At each age, the highest cross-factor correlations were between flat affect and alogia. For factors with >1 indicator, standardized factor loadings were 0.61–0.89 across ages. Explained variance in the items by the factors was 37.21–79.21%. Total variance explained by the factors across items was 62.50% at 16, 67.16.% at 17, and 56% at 22 (Supplementary table 13). Figure 1 depicts the 5-factor model at each age.

### Longitudinal Measurement Invariance

Strict invariance (constrained factor loadings, item intercepts, and residual variances) led to an unacceptable CFI change >0.01 (Supplementary table 14). Free estimation of the item 2 parameters (“my child seems emotionally ‘flat’”) resulted in an acceptable CFI change and overall fit (CFI = 0.98, RMSEA = 0.02, 90% CI 0.019, 0.022, SRMR = 0.026). This same pattern of partial strict invariance was observed in the co-twin sample (Supplementary table 15).

### Associations Between GPSs and Subdomains

Supplementary Tables 16–17 show the linear regression results from each GPS *f*. Results for the most predictive *f* are shown in table 2. Avolition showed the greatest number of significant associations across GPSs compared to the other subdomains. MDD GPS significantly predicted 4 of the 5 subdomains (*ß =* 0.041–0.084) at *q* < .05. Alogia was the only subdomain not associated with MDD GPS. The flat affect and asociality associations were not significant at 17. All associations remained significant with schizophrenia GPS as a joint predictor, except for anhedonia at 17 (Supplementary table 18). When Beta values from the MDD GPS regressions were compared (Supplementary table 19), associations were significantly stronger between MDD GPS and avolition, anhedonia, and asociality, than between MDD GPS and alogia (*P =* .008–.029), though some differences were not significant at 17.

**Table 2.** Subdomain Mean Scores Regressed on MDD GPS and Schizophrenia GPS

	*N*	MDD GPS				Schizophrenia GPS			
		*f*	*b* (SE)	*z (P)*	*β*	*f*	*b* (SE)	*z (P)*	*β*
*Age 16*									
Flat affect	6005	**0.3**	**0.019 (0.005)**	**3.504 (<.001)**	**0.05**	**1**	**0.014 (0.005)**	**2.659 (.008)**	**0.037**
Alogia	6006	1	0.010 (0.008)	1.289 (.197)	0.017	0.3	0.007 (0.008)	0.893 (.372)	0.012
Avolition	5995	**0.3**	**0.030 (0.007)**	**3.986 (<.001)**	**0.054**	0.01	–0.004 (0.008)	–0.496 (.620)	–0.007
Anhedonia	5971	**1**	**0.029 (0.009)**	**3.359 (.001)**	**0.046**	0.3	0.008 (0.009)	0.922 (.356)	0.012
Asociality	5971	**1**	**0.028 (0.006)**	**4.321 (<.001)**	**0.056**	0.01	–0.007 (0.008)	–0.858 (.391)	–0.013
*Age 17*									
Flat affect	1818	0.3	0.016 (0.012)	1.410 (.159)	0.035	1	0.027 (0.012)	2.162 (.031)	0.057
Alogia	1815	1	0.019 (0.015)	1.253 (.210)	0.03	1	0.006 (0.016)	0.387 (.699)	0.01
Avolition	1816	**1**	**0.054 (0.016)**	**3.367 (.001)**	**0.084**	**1**	**0.038 (0.016)**	**2.404 (.016)**	**0.058**
Anhedonia	1807	**1**	**0.046 (0.019)**	**2.433 (.015)**	**0.065**	0.3	0.017 (0.017)	0.967 (.334)	0.023
Asociality	1794	1	0.025 (0.016)	1.539 (.124)	0.04	0.01	–0.019 (0.016)	–1.153 (.249)	–0.03
*Age 22*									
Flat affect	6274	**0.3**	**0.018 (0.006)**	**3.195 (.001)**	**0.041**	0.01	–0.004 (0.006)	–0.737 (.461)	–0.009
Alogia	6278	1	0.009 (0.008)	1.072 (.284)	0.014	0.01	0.002 (0.008)	0.225 (.822)	0.003
Avolition	6276	**1**	**0.028 (0.008)**	**3.487 (<.001)**	**0.045**	0.01	–0.006 (0.008)	–0.738 (.460)	–0.01
Anhedonia	6251	**1**	**0.030 (0.009)**	**3.233 (.001)**	**0.043**	0.3	–0.007 (0.009)	–0.729 (.466)	–0.01
Asociality	6259	**0.3**	**0.029 (0.008)**	**3.702 (<.001)**	**0.049**	1	0.007 (0.008)	0.893 (.372)	0.011

*Note:* Subdomain mean scores regressed on MDD and schizophrenia GPS separately. Related and unrelated individuals included, using cluster-robust SE. Results shown for the most predictive GPS *f*. GPS, genome-wide polygenic score; MDD, major depressive disorder; *f*, fraction of causal markers; *b*, unstandardized regression coefficient; *β,* standardized regression coefficient. Bold typeset represents significance under corrected *q* < .05 threshold.

Significant associations were observed between schizophrenia GPS and avolition at 17 (*ß =* 0.058), and flat affect at 16 (*ß =* 0.037) at *q* < .05 (table 2). When Beta values from the regressions were compared (Supplementary table 19), the association with avolition was significantly stronger than the association with asociality at 17 (*P =* .018), and the association with flat affect was significantly stronger than the association with asociality at 16 and 17 (*P =* .035–.042). The significant associations with schizophrenia GPS did not remain significant with MDD GPS as a joint predictor (Supplementary table 18).

## Discussion

Confirmatory factor analysis was used to assess the latent structure of negative symptoms in the community in adolescence and emerging adulthood. A 5-factor model of flat affect, alogia, avolition, anhedonia, and asociality was found to fit the data best at all ages in relation to 3 other competing theory-based models and a model derived through EFA. We also found longitudinal measurement invariance of the 5-factor structure. As such, the current findings suggest that the latent structure of population-reported negative symptoms in adolescence and emerging adulthood comprises 5 dimensions and that this structure endures across time. They further suggest that the 5-factor conceptualization of negative symptoms that is empirically supported within clinical^29,30,36^ and high-risk domains^36^ generalizes beyond schizophrenia and clinical help-seeking. We found that avolition was most consistently associated with GPS for MDD and schizophrenia, in support of our hypothesis, and alogia was least associated.

The results from this study have several implications. First, they suggest that the underlying structure of negative symptoms that appears to be consistent across different stages of psychotic illness^29,30,36^ also extends to clinical populations. The current findings of inadequate fit for the 2-factor model and good fit of the 5-factor hierarchical model are further in-line with findings from clinical and high-risk samples. Previous work that has found invariance in the 5-factor structure between high-risk and first-episode psychosis samples^36^ has demonstrated that the 5-factor conceptualization is consistent across the early stages of psychotic illness. Future work will undoubtedly seek to merge data from samples at early and chronic stages of illness,^29^ and the current results lend initial support to further including community samples in such analyses. Identifying aspects of continuity or discontinuity between nonclinical, prodromal, and clinical negative symptoms may contribute to delineating the pathways involved in their development. Negative symptoms show etiological continuity across a spectrum of severity^78^ and there is evidence for some of the same genetic and environmental influences on psychotic disorders and related dimensional traits in the community.^79^ Large community samples are essential to understand the early manifestation of negative symptoms prior to illness onset and without ascertainment biases and treatment confounds inherent in clinical samples.

Second, longitudinal measurement invariance of the 5-factor structure in the general population from late adolescence to emerging adulthood suggests that the identified factor structure is not specific to a developmental age nor the result of occasion-specific properties of the measurement instrument.^80^ Collectively, these results further corroborate findings from the clinical literature that a 5-factor conceptualization of negative symptoms appears to be an empirically robust representation of the construct.

The results are the first to show associations between polygenic risk for MDD in adulthood, and avolition, flat affect, anhedonia and asociality in adolescence and emerging adulthood (*ß =* 0.041–0.084), and between polygenic risk for schizophrenia in adulthood, and avolition and flat affect in adolescence (*ß =* 0.037–0.058). The finding that avolition showed the greatest number of associations with the GPSs compared to the other subdomains may suggest that genetic vulnerability to MDD and schizophrenia could manifest particularly as avolition. The observed absence of association between MDD GPS and alogia may further provide support to suggestions that alogia may be a distinguishing feature of negative symptoms that is separable from depressive symptoms.^81,82^ However, the lack of association between schizophrenia GPS and alogia warrants further investigation. In this context, future work should ascertain whether associations between schizophrenia GPS and alogia are found at other ages across the lifespan in the general population, or whether polygenic risk for schizophrenia manifests as alogia only in clinical populations, if at all.

As well as being subdomains of the negative symptoms construct within schizophrenia, some subdomains are also core symptoms of MDD.^81–84^ This phenotypic overlap of symptoms, the genetic overlap between MDD and schizophrenia,^85^ and the high occurrence (40–80%) of depression in first-episode psychosis^86^ and schizophrenia,^87^ could be considered a challenge for understanding the etiology of negative symptoms.^81,82,88^ However, an alternative approach is to view negative symptoms within a broader, hierarchical framework of psychopathology such as the Hierarchical Taxonomy of Psychopathology (HiTOP) system,^89,90^ to harness the co-occurrence of these (and other) symptoms and traits in order to delineate etiology at multiple levels.^6,89,91^ Together with a research framework such as the NIMH Research Domain Criteria (RDoC)^92^ that seeks to understand transdiagnostic psychological processes,^93^ this is likely to be an important complement to the extant negative symptoms CFA literature in the pursuit of understanding the etiology of general and specific factors influencing both distinct and transdiagnostic symptoms.

Cumulative findings from across high-risk, clinical, and now, nonclinical domains, converge to suggest that a bifurcated conceptualization of the negative symptoms construct, as reflected in the current DSM, does not adequately capture its granularity.^27^ The genetic results presented here offer suggestive evidence to support this assertion. In the context of previous findings that have found associations between the GPSs and total negative symptoms, our results suggest that the GPSs exert symptom-level influence. The multiple-predictor GPS results add to our understanding of how MDD and schizophrenia play unique roles in influencing specific negative symptoms dimensions. Thus, our findings add to the recent shift in focus towards identifying external correlates of the 5 specific dimensions.^27,45^ They also highlight that the manifestation of polygenic liability to these psychiatric disorders may be both age-, or life stage-specific (e.g., schizophrenia GPS associations were observed in adolescence but not at age 22) as well as pervasive (i.e., MDD GPS was associated with avolition and anhedonia at all ages).

Several methodological considerations of the current study should be highlighted. Though we demonstrated pseudoreplication of the 5-factor structure, we highlight the need for replication in independent community samples and with other measures. The 5-factor model in the current study included anhedonia and asociality as single-item indicators. The use of single-item indicators in structural equation models continues to be debated,^94,95^ however, there is considerable support for their use,^96–100^ and evidence for a 5-factor structure has been found in models of negative symptoms both with and without single-item indicators in clinical samples.^29^ While it was a strength to employ parent reports and have a very large community sample, it was not feasible to collect an interview-based assessment of negative symptoms, which may have added further depth and breadth to the symptom information. Genotype data were available for approximately 60% of the sample with negative symptoms data, and the age 17 subsample was smaller than at ages 16 and 22, with only families already responding at 16 invited to participate. It is possible that parents’ time spent with their children changed across ages 16 to 22 years, and this should be considered when interpreting our results.

The 5-factor structure of negative symptoms that has been found in clinical samples also appears to be present in young people in the community. We also found dimensional associations with polygenic liability for MDD and schizophrenia, except for alogia and particularly for avolition. Our findings suggest that research into negative symptoms at the subdomain-level in the community may have the potential to inform endeavors to delineate negative symptoms beyond general population samples, both within and across diagnostic boundaries.

## Supplementary Material

Supplementary data are available at *Schizophrenia Bulletin Open* online.

Supplementary Information 1. Negative symptoms items 

Supplementary Information 2. Genotyping of individuals in the Twins Early Development Study (TEDS)

Supplementary Information 3. Calculation of genome-wide polygenic scores

Supplementary Information 4. Multiple testing correction for regression analyses

Supplementary Information 5. Models used in confirmatory factor analysis

Supplementary Information 6. Goodness-of-fit indices

Supplementary Information 7. Measurement invariance 

Supplementary Table 1. The Twins Early Development Study (TEDS) Sample

Supplementary Table 2. Parent Data Returns at Ages 16, 17 and 22 for Main and Co-Twin Samples

Supplementary Table 3. Descriptive Statistics for Negative Symptom Items, Subdomains and Totals at Ages 16, 17 and 22 in Main and Co-Twin Samples

Supplementary Table 4. Proportion of Item-Level Data Present Across Ages for Main Sample

Supplementary Table 5. Confirmatory Factor Analysis of Negative Symptoms at Ages 16, 17 and 22 in Co-Twin Sample: Model Fit Results

Supplementary Table 6. Measurement Invariance of the 5-Factor Structure of Negative Symptoms at Age 16 between the Main and Co-Twin Samples: Goodness-of-Fit Indices

Supplementary Table 7. Measurement Invariance of the 5-Factor Structure of Negative Symptoms at Age 17 between the Main and Co-Twin Samples: Goodness-of-Fit Indices

Supplementary Table 8. Measurement Invariance of the 5-Factor Structure of Negative Symptoms at Age 22 between the Main and Co-Twin Samples: Goodness-of-Fit Indices

Supplementary Table 9. Confirmatory Factor Analysis of Negative Symptoms at Ages 16, 17 and 22 in Main Sample using Diagonally Weighted Least Squares Estimation for Categorical Indicators: Model Fit Results

Supplementary Table 10. Parameter Estimates from the 5-Factor Model of Negative Symptoms at Age 16 in Main Sample

Supplementary Table 11. Parameter Estimates from the 5-Factor Model of Negative Symptoms at Age 17 in Main Sample

Supplementary Table 12. Parameter Estimates from the 5-Factor Model of Negative Symptoms at Age 22 in Main Sample

Supplementary Table 13. Communality and Uniqueness Estimates from the 5-factor Model of Negative Symptoms at Ages 16, 17 and 22 in Main Sample

Supplementary Table 14. Longitudinal Measurement Invariance of the 5-Factor Structure of Negative Symptoms between Ages 16, 17 and 22 in the Main Sample: Goodness-of-Fit Indices

Supplementary Table 15. Longitudinal Measurement Invariance of the 5-Factor Structure of Negative Symptoms between Ages 16, 17 and 22 in the Co-Twin Sample: Goodness-of-Fit Indices

Supplementary Table 16. Linear Regression Results for Subdomain Mean Scores Regressed on Major Depressive Disorder GPS

Supplementary Table 17. Linear Regression Results for Subdomain Mean Scores Regressed on Schizophrenia GPS

Supplementary Table 18. Multiple Linear Regression Results for Subdomain Mean Scores Regressed on Major Depressive Disorder GPS and Schizophrenia GPS

Supplementary Table 19. Pairwise Wald Test Results for Subdomain Mean Scores Regressed on Major Depressive Disorder GPS and Schizophrenia GPS

Supplementary Figure 1. Plot of the First and Second Principal Components of Ancestry

Supplementary Figure 2. Plots of Subdomain Mean Scores by Major Depressive Disorder GPS Decile Group

Supplementary Figure 3. Plots of Subdomain Mean Scores by Schizophrenia GPS Decile Group

sgac009_suppl_Supplementary_Data

## References

[1] Patel R , Jayatilleke N, Broadbent M, et al. Negative symptoms in schizophrenia: a study in a large clinical sample of patients using a novel automated method. BMJ Open. 2015;5(9):e007619. 10.1136/bmjopen-2015-007619 PMC4577949 26346872

[2] Rabinowitz J , Berardo CG, Bugarski-Kirola D, Marder S. Association of prominent positive and prominent negative symptoms and functional health, well-being, healthcare-related quality of life and family burden: a CATIE analysis. Schizophr Res. 2013;150(2-3):339–342. 23899997 10.1016/j.schres.2013.07.014

[3] Ho BC , Nopoulos P, Flaum M, Arndt S, Andreasen NC. Two-year outcome in first-episode schizophrenia: predictive value of symptoms for quality of life. Am J Psychiatry. 1998;155(9):1196–1201. 9734542 10.1176/ajp.155.9.1196

[4] Rabinowitz J , Levine SZ, Garibaldi G, Bugarski-Kirola D, Berardo CG, Kapur S. Negative symptoms have greater impact on functioning than positive symptoms in schizophrenia: analysis of CATIE data. Schizophr Res. 2012;137(1-3):147–150. 22316568 10.1016/j.schres.2012.01.015

[5] Piskulic D , Addington J, Cadenhead KS, et al. Negative symptoms in individuals at clinical high risk of psychosis. Psychiatry Res. 2012;196(2-3):220–224. 22445704 10.1016/j.psychres.2012.02.018 PMC4119605

[6] Cowan HR , Mittal VA. Transdiagnostic dimensions of psychiatric comorbidity in individuals at clinical high risk for psychosis: a preliminary study informed by HiTOP. Front Psychiatry. 2020;11:614710. 33488432 10.3389/fpsyt.2020.614710 PMC7819881

[7] Velthorst E , Nieman DH, Becker HE, et al. Baseline differences in clinical symptomatology between ultra high risk subjects with and without a transition to psychosis. Schizophr Res. 2009;109(1-3):60–65. 19272756 10.1016/j.schres.2009.02.002

[8] an der Heiden W , Häfner H. The epidemiology of onset and course of schizophrenia. Eur Arch Psychiatry Clin Neurosci. 2000;250(6):292–303. 11153964 10.1007/s004060070004

[9] Catalan A , Salazar de Pablo G, Vaquerizo Serrano J, et al. Annual Research Review: prevention of psychosis in adolescents—systematic review and meta-analysis of advances in detection, prognosis and intervention. J Child Psychol Psychiatry. 2021;62(5):657–673. 32924144 10.1111/jcpp.13322

[10] Lyne J , O’Donoghue B, Roche E, Renwick L, Cannon M, Clarke M. Negative symptoms of psychosis: a life course approach and implications for prevention and treatment. Early Interv Psychiatry. 2018;12(4):561–571. 29076240 10.1111/eip.12501

[11] van Os J , Guloksuz S. A critique of the “ultra-high risk” and “transition” paradigm. World Psychiatry. 2017;16(2):200–206. 28498576 10.1002/wps.20423 PMC5428198

[12] Gupta T , Cowan HR, Strauss GP, Walker EF, Mittal VA. Deconstructing negative symptoms in individuals at clinical high-risk for psychosis: evidence for volitional and diminished emotionality subgroups that predict clinical presentation and functional outcome. Schizophr Bull. 2021;47(1):54–63. doi:10.1093/schbul/sbaa084 32955097 PMC7825091

[13] Lyne J , Renwick L, Madigan K, et al. Do psychosis prodrome onset negative symptoms predict first presentation negative symptoms? Eur Psychiatry. 2014;29(3):153–159. 23523737 10.1016/j.eurpsy.2013.02.003

[14] Yung AR , Nelson B, McGorry PD, Wood SJ, Lin A. Persistent negative symptoms in individuals at Ultra High Risk for psychosis. Schizophr Res. 2019;206:355–361. 30482643 10.1016/j.schres.2018.10.019 PMC6542412

[15] Ronald A , Sieradzka D, Cardno AG, Haworth CM, McGuire P, Freeman D. Characterization of psychotic experiences in adolescence using the specific psychotic experiences questionnaire: findings from a study of 5000 16-year-old twins. Schizophr Bull. 2014;40(4):868–877. 24062593 10.1093/schbul/sbt106 PMC4059437

[16] Barragan M , Laurens KR, Navarro JB, Obiols JE. Psychotic-like experiences and depressive symptoms in a community sample of adolescents. Eur Psychiatry. 2011;26(6):396–401. 21334860 10.1016/j.eurpsy.2010.12.007

[17] Kessler RC , Amminger GP, Aguilar-Gaxiola S, Alonso J, Lee S, Ustün TB. Age of onset of mental disorders: a review of recent literature. Curr Opin Psychiatry. 2007;20(4):359–364. 17551351 10.1097/YCO.0b013e32816ebc8c PMC1925038

[18] Maki PH , Miettunen J, Kaakinen M, et al. P0164—Negative symptoms precede the onset of first episode psychosis in a prospective general population sample of adolescents. Eur Psychiatry. 2008;23(S2):S129–S129.

[19] Dominguez MD , Saka MC, can Saka M, Lieb R, Wittchen HU, van Os J. Early expression of negative/disorganized symptoms predicting psychotic experiences and subsequent clinical psychosis: a 10-year study. Am J Psychiatry. 2010;167(9):1075–1082. 20634371 10.1176/appi.ajp.2010.09060883

[20] Werbeloff N , Dohrenwend BP, Yoffe R, van Os J, Davidson M, Weiser M. The association between negative symptoms, psychotic experiences and later schizophrenia: a population-based longitudinal study. PLoS One. 2015;10(3):e0119852. 25748557 10.1371/journal.pone.0119852 PMC4351950

[21] Correll CU , Schooler NR. Negative symptoms in schizophrenia: a review and clinical guide for recognition, assessment, and treatment. Neuropsychiatr Dis Treat. 2020;16:519–534. 32110026 10.2147/NDT.S225643 PMC7041437

[22] Fusar-Poli P , Papanastasiou E, Stahl D, et al. Treatments of negative symptoms in schizophrenia: meta-analysis of 168 randomized placebo-controlled trials. Schizophr Bull. 2015;41(4):892–899. 25528757 10.1093/schbul/sbu170 PMC4466178

[23] Kantrowitz JT . How do we address treating the negative symptoms of schizophrenia pharmacologically? Expert Opin Pharmacother. 2021;22(14):1811–1813. doi:10.1080/14656566.2021.1939677 34130578

[24] Aleman A , Lincoln TM, Bruggeman R, et al. Treatment of negative symptoms: where do we stand, and where do we go? Schizophr Res. 2017;186:55–62. 27293137 10.1016/j.schres.2016.05.015

[25] Krause M , Zhu Y, Huhn M, et al. Antipsychotic drugs for patients with schizophrenia and predominant or prominent negative symptoms: a systematic review and meta-analysis. Eur Arch Psychiatry Clin Neurosci. 2018;268(7):625–639. 29368205 10.1007/s00406-018-0869-3

[26] Kirkpatrick B , Fenton WS, Carpenter WT Jr, Marder SR. The NIMH-MATRICS consensus statement on negative symptoms. Schizophr Bull. 2006;32(2):214–219. 16481659 10.1093/schbul/sbj053 PMC2632223

[27] Strauss GP , Ahmed AO, Young JW, Kirkpatrick B. Reconsidering the latent structure of negative symptoms in schizophrenia: a review of evidence supporting the 5 consensus domains. Schizophr Bull. 2019;45(4):725–729. 30541136 10.1093/schbul/sby169 PMC6581128

[28] Messinger JW , Trémeau F, Antonius D, et al. Avolition and expressive deficits capture negative symptom phenomenology: implications for DSM-5 and schizophrenia research. Clin Psychol Rev. 2011;31(1):161–168. 20889248 10.1016/j.cpr.2010.09.002 PMC2997909

[29] Strauss GP , Nuñez A, Ahmed AO, et al. The latent structure of negative symptoms in schizophrenia. JAMA Psychiatry. 2018;75(12):1271–1279. 30208377 10.1001/jamapsychiatry.2018.2475 PMC6583036

[30] Ahmed AO , Kirkpatrick B, Galderisi S, et al. Cross-cultural validation of the 5-factor structure of negative symptoms in schizophrenia. Schizophr Bull. 2019;45(2):305–314. 29912473 10.1093/schbul/sby050 PMC6403061

[31] Lenzenweger MF , Dworkin RH, Wethington E. Models of positive and negative symptoms in schizophrenia: an empirical evaluation of latent structures. J Abnorm Psychol. 1989;98(1):62–70. 2708643 10.1037//0021-843x.98.1.62

[32] Lenzenweger MF , Dworkin RH, Wethington E. Examining the underlying structure of schizophrenic phenomenology: evidence for a three-process model. Schizophr Bull. 1991;17(3):515–524. 1947875 10.1093/schbul/17.3.515

[33] Strauss GP , Esfahlani FZ, Galderisi S, et al. Network analysis reveals the latent structure of negative symptoms in schizophrenia. Schizophr Bull. 2019;45(5):1033–1041. 30256991 10.1093/schbul/sby133 PMC6737465

[34] Galderisi S , Kaiser S, Bitter I, et al. EPA guidance on treatment of negative symptoms in schizophrenia. Eur Psychiatry. 2021;64(1):e21. 33726883 10.1192/j.eurpsy.2021.13 PMC8057437

[35] Mucci A , Vignapiano A, Bitter I, et al. A large European, multicenter, multinational validation study of the Brief Negative Symptom Scale. Eur Neuropsychopharmacol. 2019;29(8):947–959. 31255394 10.1016/j.euroneuro.2019.05.006

[36] Chang WC , Strauss GP, Ahmed AO, et al. The latent structure of negative symptoms in individuals with attenuated psychosis syndrome and early psychosis: support for the 5 consensus domains. Schizophr Bull. 2021;47(2):386–394. 32909606 10.1093/schbul/sbaa129 PMC7965067

[37] Ang MS , Rekhi G, Lee J. Validation of the Brief Negative Symptom Scale and its association with functioning. Schizophr Res. 2019;208:97–104. 30987926 10.1016/j.schres.2019.04.005

[38] Rodríguez-Testal JF , Perona-Garcelán S, Dollfus S, et al. Spanish validation of the self-evaluation of negative symptoms scale SNS in an adolescent population. BMC Psychiatry. 2019;19(1):327. 31664965 10.1186/s12888-019-2314-1 PMC6819523

[39] Stefanis NC , Hanssen M, Smirnis NK, et al. Evidence that three dimensions of psychosis have a distribution in the general population. Psychol Med. 2002;32(2):347–358. 11866327 10.1017/s0033291701005141

[40] Ziermans TB . Working memory capacity and psychotic-like experiences in a general population sample of adolescents and young adults. Front Psychiatry. 2013;4:161. 24348432 10.3389/fpsyt.2013.00161 PMC3847810

[41] Liu J , Wong KK, Dong F, Raine A, Tuvblad C. The Schizotypal Personality Questionnaire - Child (SPQ-C): psychometric properties and relations to behavioral problems with multi-informant ratings. Psychiatry Res. 2019;275:204–211. 30928723 10.1016/j.psychres.2019.03.006 PMC6748384

[42] Raine A , Wong KKY, Liu J. The Schizotypal Personality Questionnaire for Children (SPQ-C): factor structure, child abuse, and family history of schizotypy. Schizophr Bull. 2021;47(2):323–331. doi:10.1093/schbul/sbaa100 32674122 10.1093/schbul/sbaa100 PMC8370046

[43] Fonseca-Pedrero E , Ortuño-Sierra J, Lucas-Molina B, et al. Brief assessment of schizotypal traits: a multinational study. Schizophr Res. 2018;197:182–191. 29113776 10.1016/j.schres.2017.10.043

[44] Foussias G , Siddiqui I, Fervaha G, Agid O, Remington G. Dissecting negative symptoms in schizophrenia: opportunities for translation into new treatments. J Psychopharmacol. 2015;29(2):116–126. 25516370 10.1177/0269881114562092

[45] Galderisi S , Mucci A, Buchanan RW, Arango C. Negative symptoms of schizophrenia: new developments and unanswered research questions. Lancet Psychiatry. 2018;5(8):664–677. 29602739 10.1016/S2215-0366(18)30050-6

[46] Xavier RM , Vorderstrasse A. genetic basis of positive and negative symptom domains in schizophrenia. Biol Res Nurs. 2017;19(5):559–575. 28691507 10.1177/1099800417715907

[47] Marder SR , Galderisi S. The current conceptualization of negative symptoms in schizophrenia. World Psychiatry. 2017;16(1):14–24. 28127915 10.1002/wps.20385 PMC5269507

[48] Shaffer JJ , Peterson MJ, McMahon MA, et al. Neural correlates of schizophrenia negative symptoms: distinct subtypes impact dissociable brain circuits. Mol Neuropsychiatry. 2015;1(4):191–200. 27606313 10.1159/000440979 PMC4996000

[49] Hanssen E , Krabbendam L, Robberegt S, Fett AK. Social and non-social reward learning reduced and related to a familial vulnerability in schizophrenia spectrum disorders. Schizophr Res. 2020;215:256–262. 31753593 10.1016/j.schres.2019.10.019

[50] Hanssen EMs , van der Velde JP, Gromann PMs, et al. Neural correlates of reward processing in healthy siblings of patients with schizophrenia. Front Hum Neurosci. 2015;9:504. doi:10.3389/fnhum.2015.00504 26441601 PMC4585217

[51] Strauss GP , Waltz JA, Gold JM. A review of reward processing and motivational impairment in schizophrenia. Schizophr Bull. 2014;40 Suppl 2:S107–S116. 24375459 10.1093/schbul/sbt197 PMC3934394

[52] Xu C , Aragam N, Li X, et al. BCL9 and C9orf5 are associated with negative symptoms in schizophrenia: meta-analysis of two genome-wide association studies. PLoS One. 2013;8(1):e51674. 23382809 10.1371/journal.pone.0051674 PMC3558516

[53] Bigdeli TB , Peterson RE, Ripke S, et al. Genome-wide association study of clinical features in the schizophrenia psychiatric genomics consortium: confirmation of polygenic effect on negative symptoms. bioRxiv. Published online July 9, 2017:161349. doi:10.1101/161349

[54] Fanous AH , Zhou B, Aggen SH, et al.; Schizophrenia Psychiatric Genome-Wide Association Study (GWAS) Consortium. Genome-wide association study of clinical dimensions of schizophrenia: polygenic effect on disorganized symptoms. Am J Psychiatry. 2012;169(12):1309–1317. 23212062 10.1176/appi.ajp.2012.12020218 PMC3646712

[55] Xavier RM , Dungan JR, Keefe RSE, Vorderstrasse A. Polygenic signal for symptom dimensions and cognitive performance in patients with chronic schizophrenia. Schizophr Res Cogn. 2018;12:11–19. 29552508 10.1016/j.scog.2018.01.001 PMC5852279

[56] Sengupta SM , MacDonald K, Fathalli F, et al. Polygenic Risk Score associated with specific symptom dimensions in first-episode psychosis. Schizophr Res. 2017;184:116–121. 27916287 10.1016/j.schres.2016.11.039

[57] Pain O , Dudbridge F, Cardno AG, et al. Genome-wide analysis of adolescent psychotic-like experiences shows genetic overlap with psychiatric disorders. Am J Med Genet B Neuropsychiatr Genet. 2018;177(4):416–425. 29603866 10.1002/ajmg.b.32630 PMC6001485

[58] Jones HJ , Stergiakouli E, Tansey KE, et al. Phenotypic manifestation of genetic risk for schizophrenia during adolescence in the general population. JAMA Psychiatry. 2016;73(3):221–228. 26818099 10.1001/jamapsychiatry.2015.3058 PMC5024747

[59] Foussias G , Remington G. Negative symptoms in schizophrenia: avolition and Occam’s razor. Schizophr Bull. 2010;36(2):359–369. 18644851 10.1093/schbul/sbn094 PMC2833114

[60] Strauss GP , Bartolomeo LA, Harvey PD. Avolition as the core negative symptom in schizophrenia: relevance to pharmacological treatment development. NPJ Schizophr. 2021;7(1):16. 33637748 10.1038/s41537-021-00145-4 PMC7910596

[61] Strauss GP , Zamani Esfahlani F, Sayama H, et al. Network analysis indicates that avolition is the most central domain for the successful treatment of negative symptoms: evidence from the roluperidone randomized clinical trial. Schizophr Bull. Published online 2020;46(4):964–970. doi:10.1093/schbul/sbz141 31989151 PMC7342174

[62] Haworth CM , Davis OS, Plomin R. Twins Early Development Study (TEDS): a genetically sensitive investigation of cognitive and behavioral development from childhood to young adulthood. Twin Res Hum Genet. 2013;16(1):117–125. 23110994 10.1017/thg.2012.91 PMC3817931

[63] TEDS Data Dictionary. Accessed December 16, 2020. https://www.teds.ac.uk/datadictionary/

[64] Andreasen NC . Negative symptoms in schizophrenia. Definition and reliability. Arch Gen Psychiatry. 1982;39(7):784–788. 7165477 10.1001/archpsyc.1982.04290070020005

[65] Selzam S , McAdams TA, Coleman JRI, et al. Evidence for gene-environment correlation in child feeding: links between common genetic variation for BMI in children and parental feeding practices. PLoS Genet. 2018;14(11):e1007757. 30457987 10.1371/journal.pgen.1007757 PMC6245504

[66] Choi SW , Mak TS, O’Reilly PF. Tutorial: a guide to performing polygenic risk score analyses. Nat Protoc. 2020;15(9):2759–2772. 32709988 10.1038/s41596-020-0353-1 PMC7612115

[67] Selzam S , Ritchie SJ, Pingault JB, Reynolds CA, O’Reilly PF, Plomin R. Comparing within- and between-family polygenic score prediction. Am J Hum Genet. 2019;105(2):351–363. 31303263 10.1016/j.ajhg.2019.06.006 PMC6698881

[68] Vilhjálmsson BJ , Yang J, Finucane HK, et al.; Schizophrenia Working Group of the Psychiatric Genomics Consortium, Discovery, Biology, and Risk of Inherited Variants in Breast Cancer (DRIVE) study. Modeling linkage disequilibrium increases accuracy of polygenic risk scores. Am J Hum Genet. 2015;97(4):576–592. 26430803 10.1016/j.ajhg.2015.09.001 PMC4596916

[69] Wray NR , Ripke S, Mattheisen M, et al.; eQTLGen; 23andMe; Major Depressive Disorder Working Group of the Psychiatric Genomics Consortium. Genome-wide association analyses identify 44 risk variants and refine the genetic architecture of major depression. Nat Genet. 2018;50(5):668–681. 29700475 10.1038/s41588-018-0090-3 PMC5934326

[70] Pardiñas AF , Holmans P, Pocklington AJ, et al.; GERAD1 Consortium; CRESTAR Consortium. Common schizophrenia alleles are enriched in mutation-intolerant genes and in regions under strong background selection. Nat Genet. 2018;50(3):381–389. 29483656 10.1038/s41588-018-0059-2 PMC5918692

[71] Hu L tze , Bentler PM. Cutoff criteria for fit indexes in covariance structure analysis: Conventional criteria versus new alternatives. Struct Equ Model Multidiscip J. 1999;6(1):1–55.

[72] Neath AA , Cavanaugh JE. The Bayesian information criterion: background, derivation, and applications. Wiley Interdiscip Rev Comput Stat. 2012;4(2):199–203.

[73] Burnham KP , Anderson DR. Multimodel inference: understanding AIC and BIC in model selection. Sociol Methods Res. 2004;33(2):261–304.

[74] Chen FF . Sensitivity of goodness of fit indexes to lack of measurement invariance. Struct Equ Model Multidiscip J. 2007;14(3):464–504.

[75] Rosseel Y . lavaan: An R package for structural equation modeling. J Stat Softw. 2012;48(2):1–36.

[76] Benjamini Y , Hochberg Y. Controlling the false discovery rate: a practical and powerful approach to multiple testing. J R Stat Soc Ser B Methodol. 1995;57(1):289–300.

[77] Klopp E . A tutorial on testing the equality of standardized regression coefficients in structural equation models using Wald tests with lavaan. Published online 2019. https://scholar.google.co.uk/scholar?hl=en&as_sdt=0%2C5&q=A+Tutorial+on+Testing+the+Equality+of+Standardized+Regression+Coefficients+in+Structural+Equation+Models+using+Wald+Tests+with+lavaan&btnG=

[78] Zavos HM , Freeman D, Haworth CM, et al. Consistent etiology of severe, frequent psychotic experiences and milder, less frequent manifestations: a twin study of specific psychotic experiences in adolescence. JAMA Psychiatry. 2014;71(9):1049–1057. 25075799 10.1001/jamapsychiatry.2014.994 PMC4156464

[79] Ronald A , Pain O. A systematic review of genome-wide research on psychotic experiences and negative symptom traits: new revelations and implications for psychiatry. Hum Mol Genet. 2018;27(R2):R136–R152. 29741616 10.1093/hmg/ddy157 PMC6061705

[80] Grimm KJ , Ram N, Estabrook R. Growth Modeling: Structural Equation and Multilevel Modeling Approaches. New York: The Guildford Press; 2017.

[81] Strauss GP , Cohen AS. A transdiagnostic review of negative symptom phenomenology and etiology. Schizophr Bull. 2017;43(4):712–719. 28969356 10.1093/schbul/sbx066 PMC5472109

[82] Krynicki CR , Upthegrove R, Deakin JFW, Barnes TRE. The relationship between negative symptoms and depression in schizophrenia: a systematic review. Acta Psychiatr Scand. 2018;137(5):380–390. 29532909 10.1111/acps.12873

[83] Edwards CJ , Garety P, Hardy A. The relationship between depressive symptoms and negative symptoms in people with non-affective psychosis: a meta-analysis. Psychol Med. 2019;49(15):2486–2498. 31530319 10.1017/S0033291719002381

[84] Rosebrock LE , Waite F, Diamond R, et al. Anticipatory pleasure in current psychosis: Cognitive and emotional correlates. Psychiatry Res. 2021;297:113697. 33465523 10.1016/j.psychres.2020.113697

[85] Lee SH , Ripke S, Neale BM, et al. Genetic relationship between five psychiatric disorders estimated from genome-wide SNPs. Nat Genet. 2013;45(9):984–994. 23933821 10.1038/ng.2711 PMC3800159

[86] Upthegrove R , Birchwood M, Ross K, Brunett K, McCollum R, Jones L. The evolution of depression and suicidality in first episode psychosis. Acta Psychiatr Scand. 2010;122(3):211–218. 19922525 10.1111/j.1600-0447.2009.01506.x

[87] Conley RR , Ascher-Svanum H, Zhu B, Faries DE, Kinon BJ. The burden of depressive symptoms in the long-term treatment of patients with schizophrenia. Schizophr Res. 2007;90(1-3):186–197. 17110087 10.1016/j.schres.2006.09.027 PMC1937504

[88] Upthegrove R , Marwaha S, Birchwood M. Depression and schizophrenia: cause, consequence, or trans-diagnostic issue? Schizophr Bull. 2017;43(2):240–244. 27421793 10.1093/schbul/sbw097 PMC5605248

[89] Kotov R , Jonas KG, Carpenter WT, et al.; HiTOP Utility Workgroup. Validity and utility of Hierarchical Taxonomy of Psychopathology (HiTOP): I. Psychosis superspectrum. World Psychiatry. 2020;19(2):151–172. 32394571 10.1002/wps.20730 PMC7214958

[90] Kotov R , Krueger RF, Watson D. A paradigm shift in psychiatric classification: the Hierarchical Taxonomy Of Psychopathology (HiTOP). World Psychiatry. 2018;17(1):24–25. 29352543 10.1002/wps.20478 PMC5775140

[91] Waszczuk MA , Eaton NR, Krueger RF, et al. Redefining phenotypes to advance psychiatric genetics: Implications from hierarchical taxonomy of psychopathology. J Abnorm Psychol. 2020;129(2):143–161. 31804095 10.1037/abn0000486 PMC6980897

[92] Insel T , Cuthbert B, Garvey M, et al. Research domain criteria (RDoC): toward a new classification framework for research on mental disorders. Am J Psychiatry. 2010;167(7):748–751. 20595427 10.1176/appi.ajp.2010.09091379

[93] Cuthbert BN . The RDoC framework: facilitating transition from ICD/DSM to dimensional approaches that integrate neuroscience and psychopathology. World Psychiatry. 2014;13(1):28–35. 24497240 10.1002/wps.20087 PMC3918011

[94] Hayduk LA , Littvay L. Should researchers use single indicators, best indicators, or multiple indicators in structural equation models? BMC Med Res Methodol. 2012;12:159. 23088287 10.1186/1471-2288-12-159 PMC3506474

[95] Petrescu M . Marketing research using single-item indicators in structural equation models. J Mark Anal. 2013;1(2):99–117.

[96] Gosling SD , Rentfrow PJ, Swann Jr WB. A very brief measure of the Big-Five personality domains. J Res Personal. 2003;37(6):504–528.

[97] Benet-Martínez V , Leu J, Lee F, Morris MW. Negotiating biculturalism: cultural frame switching in biculturals with oppositional versus compatible cultural identities. J Cross-Cult Psychol. 2002;33(5):492–516.

[98] Robins RW , Hendin HM, Trzesniewski KH. Measuring global self-esteem: construct validation of a single-item measure and the Rosenberg Self-Esteem Scale. Pers Soc Psychol Bull. 2001;27(2):151–161.

[99] Robins RW , Tracy JL, Trzesniewski K, Potter J, Gosling SD. Personality correlates of self-esteem. J Res Personal. 2001;35(4):463–482.

[100] Postmes T , Haslam SA, Jans L. A single-item measure of social identification: reliability, validity, and utility. Br J Soc Psychol. 2013;52(4):597–617. 23121468 10.1111/bjso.12006

