# Supplementary material for: The Latent Structure of Negative Symptoms in the General Population in Adolescence and Emerging Adulthood
Source: Schizophr Bull Open. 2022 Jan 12;3(1):sgac009. doi: 10.1093/schizbullopen/sgac009 (PMC8827402; doi:10.1093/schizbullopen/sgac009)
Supplement: sgac009_suppl_Supplementary_Data [file sgac009_suppl_Supplementary_Data.pdf]

The Latent Structure of Negative Symptoms in the General Population in Adolescence and Emerging Adulthood

Supplementary Material. Contents

Supplementary Information 1. Negative symptoms items
Supplementary Information 2. Genotyping of individuals in the Twins Early Development Study (TEDS)
Supplementary Information 3. Calculation of genome-wide polygenic scores
Supplementary Information 4. Multiple testing correction for regression analyses
Supplementary Information 5. Models used in confirmatory factor analysis
Supplementary Information 6. Goodness-of-fit indices
Supplementary Information 7. Measurement invariance
Supplementary Table 1. The Twins Early Development Study (TEDS) Sample
Supplementary Table 2. Parent Data Returns at Ages 16, 17 and 22 for Main and Co-Twin Samples
Supplementary Table 3. Descriptive Statistics for Negative Symptom Items, Subdomains and Totals at Ages 16, 17 and 22 in Main and Co-Twin Samples
Supplementary Table 4. Proportion of Item-Level Data Present Across Ages for Main Sample
Supplementary Table 5. Confirmatory Factor Analysis of Negative Symptoms at Ages 16, 17 and 22 in Co-Twin Sample: Model Fit Results
Supplementary Table 6. Measurement Invariance of the 5-Factor Structure of Negative Symptoms at Age 16 between the Main and Co-Twin Samples: Goodness-of-Fit Indices
Supplementary Table 7. Measurement Invariance of the 5-Factor Structure of Negative Symptoms at Age 17 between the Main and Co-Twin Samples: Goodness-of-Fit Indices
Supplementary Table 8. Measurement Invariance of the 5-Factor Structure of Negative Symptoms at Age 22 between the Main and Co-Twin Samples: Goodness-of-Fit Indices
Supplementary Table 9. Confirmatory Factor Analysis of Negative Symptoms at Ages 16, 17 and 22 in Main Sample using Diagonally Weighted Least Squares Estimation for Categorical Indicators: Model Fit Results
Supplementary Table 10. Parameter Estimates from the 5-Factor Model of Negative Symptoms at Age 16 in Main Sample
Supplementary Table 11. Parameter Estimates from the 5-Factor Model of Negative Symptoms at Age 17 in Main Sample
Supplementary Table 12. Parameter Estimates from the 5-Factor Model of Negative Symptoms at Age 22 in Main Sample
Supplementary Table 13. Communality and Uniqueness Estimates from the 5-factor Model of Negative Symptoms at Ages 16, 17 and 22 in Main Sample
Supplementary Table 14. Longitudinal Measurement Invariance of the 5-Factor Structure of Negative Symptoms between Ages 16, 17 and 22 in the Main Sample: Goodness-of-Fit Indices
Supplementary Table 15. Longitudinal Measurement Invariance of the 5-Factor Structure of Negative Symptoms between Ages 16, 17 and 22 in the Co-Twin Sample: Goodness-of-Fit Indices
Supplementary Table 16. Linear Regression Results for Subdomain Mean Scores Regressed on Major Depressive Disorder GPS
Supplementary Table 17. Linear Regression Results for Subdomain Mean Scores Regressed on Schizophrenia GPS
Supplementary Table 18. Multiple Linear Regression Results for Subdomain Mean Scores Regressed on Major Depressive Disorder GPS and Schizophrenia GPS
Supplementary Table 19. Pairwise Wald Test Results for Subdomain Mean Scores Regressed on Major Depressive Disorder GPS and Schizophrenia GPS
Supplementary Figure 1. Plot of the First and Second Principal Components of Ancestry
Supplementary Figure 2. Plots of Subdomain Mean Scores by Major Depressive Disorder GPS Decile Group
Supplementary Figure 3. Plots of Subdomain Mean Scores by Schizophrenia GPS Decile Group

Supplementary Information 1. Negative symptoms items

Parents were asked, for each twin separately, to rate how strongly they agree or disagree (‘not at all’, ‘somewhat true’, ‘mainly true’, ‘definitely true’) with the following statements:

1. My child often fails to smile or laugh at things others would find funny
2. My child seems emotionally ‘flat’, for example, rarely changes the emotions he/she shows
3. My child usually gives brief, one word replies to questions, even if encouraged to say more
4. My child often does not have much to say for himself/herself
5. My child often sits around for a long time doing nothing
6. My child has a lack of energy and motivation
7. My child has very few interests or hobbies
8. My child has few or no friends

The following 2 items relating to attention were not included in the current analyses:

9. My child does not pay attention when being spoken to
10. My child is often inattentive and appears distracted

Supplementary Information 2. Genotyping of individuals in the Twins Early Development Study (TEDS)

Full details of the genotyping procedures can be found on the TEDS data dictionary website (https://www.teds.ac.uk/datadictionary/studies/dna.htm). There have been 5 phases of genotyping in the TEDS sample since 1998. Data from all phases has contributed towards the ‘genotypic sample’ in TEDS, for which genome-wide polygenic scores were calculated. DNA was collected from cheek swabs between 1998 and 2009 for phases 1-4, and from saliva samples between 2014-2015 for phase 5. Twin pairs (or individual twins) who had recently returned data were prioritised for DNA collection. Families were contacted by mail in phase 1. In phase 2, families were contacted by phone before by mail, following initial verbal consent. In the later phases, families were contacted by mail followed by phone for families who had not responded. Cheek swab samples were collected from individuals by their parents and saliva samples were collected by individuals themselves. Collection was carried out at home and samples were returned by post. The Affymetrix platform was used for the cheek swab samples from phases 1-4 (AffymetrixGeneChip 6.0 SNP arrays). The Illumina Human OEE platform was used for the saliva samples from phase 5 (using OmniExpressExome-8v1.2 arrays). The OEE platform was also used for some cheek swab samples from earlier phases (see https://www.teds.ac.uk/datadictionary/studies/dna.htm#oee). Detailed information regarding exclusions can be found on the TEDS data dictionary website (https://www.teds.ac.uk/datadictionary/studies/dna.htm); broad exclusions were made on the basis that parents self-reported their ethnic origin as ‘other’ than ‘white’, and where serious medical conditions and or perinatal complications had been self-reported.

The genotypic sample in TEDS includes data from both the Affymetrix and OEE platforms, which were combined and subjected to quality control procedures (described in detail in S1 Methods, Supplementary Methods, Selzam et al., 2018). From an initial combined sample size of 11869, 1523 samples were removed owing to possible non-European ancestry, heterozygosity anomalies, genotype call rate <0.98, and genetic relatedness other than dyzygosity. The final genotypic sample is comprised of 10346 individual twins (3057 genotyped on Affymetrix, 7289 genotyped on OEE). Of the 10346 individuals, there is genotype data from 3320 dyzygotic twin pairs. There are 3706 twin pairs of any zygosity with only 1 twin genotyped (2666 monozygotic, 1017 dyzygotic and 23 unknown zygosity). There are 7026 twin pairs with either 1 or both twin genotyped. Seven million (7)363646 genotyped and imputed single nucleotide polymorphisms (SNPs) were retained for subsequent analyses.

References

Selzam S, McAdams TA, Coleman JRI, et al. Evidence for gene-environment correlation in child feeding: Links between common genetic variation for BMI in children and parental feeding practices. *PLoS Genet*. 2018;14(11). doi:10.1371/journal.pgen.1007757

Supplementary Information 3. Calculation of genome-wide polygenic scores

Genome-wide polygenic scores (GPS) were calculated for each of the 10346 individuals in the genotypic sample (see Supplementary Information 2). GPS for MDD were derived using data from the 2018 GWAS (genome-wide association study) meta-analysis (with 23andMe samples removed), comprising 75607 cases and 231747 controls (Wray et al., 2018). GPS for schizophrenia were derived using data from the 2018 GWAS, comprising 40675 cases and 64643 controls (Pardiñas et al., 2018). The description (below) of the methods used for the GPS calculation is adapted directly from Selzam et al. (2019; Supplementary Methods), where the methods are fully described.

GPS are the sum of single nucleotide polymorphisms (SNPs), individual genetic variants, associated with an outcome that are carried by an individual, weighted by the effect sizes of the SNPs. SNP-effect sizes are estimated in a genome-wise association study (GWAS) of an outcome of interest in an independent sample, in which individual SNPs are regressed on the outcome. LDpred software (Vilhjálmsson et al., 2015) was used to calculate the GPS. LDpred implements Bayesian methods, adjusting for linkage disequilibrium (LD) amongst SNPs rather than removing SNPs that are in high LD (as is the case with the clumping and thresholding approach, see, e.g., Choi et al., 2020). LDpred estimates a posterior effect size for each SNP that is present in the GWAS summary statistics as well as in the (target) genotyped sample.

The posterior effect size is estimated as the original summary statistic effect size estimate, adjusted by the relative influence of a SNP (taking into account its level of LD with surrounding SNPs in the target sample) and adjusting for a prior on the effect size of each SNP. A radius corresponding to a 2 megabase window on average around each SNP of interest was set to account for LD. The effect size prior is dependent on the SNP-heritability of the GWAS outcome of interest, and the proportion of SNPs (the fraction of causal markers) believed to influence the outcome. Using the effect size prior, the beta effect sizes are reweighted. Thus, the effects are spread among the SNPs across the genome in proportion to the amount of LD amongst them. The genotype dataset was reduced to SNPs that had imputation quality information scores of 1 to reduce computational demands, resulting in 515100 SNPs that could be analysed. Alleles associated with the outcome were counted for each individual (0, 1, or 2 for each SNP). The GPS for each individual was calculated as the sum of the alleles, each weighted by the posterior SNP effect size.

The first 10 principal components (PCs) were calculated using data from the final genotyped sample, and GPSs were regressed on these PCs prior to analysis. These PCs reflect and capture population structure within the sample. Regressing the GPSs on the principal components adjusts for any confounding that would otherwise be present due to population structure. GPSs were also regressed on batch and chip type to further remove any potential confounding by these variables. Standardized residuals were used in the GPS analyses.

Supplementary Information 3. Calculation of genome-wide polygenic scores

Genotype data from individuals with parent-report data (at age 16, 17 or 22) was used in the calculation of GPS deciles. These were calculated for the most predictive fraction of causal markers for each subdomain at each age (see Supplementary Tables 15 and 16). GPS decile plots are shown in Supplementary Figures 2 and 3.

References

Choi, S. W., Mak, T. S. H., O'Reilly, P. F. Tutorial: a guide to performing polygenic risk score analyses. *Nature Protocols*. 2020;15(9):2759-2772.

Pardiñas AF, Holmans P, Pocklington AJ, et al. Common schizophrenia alleles are enriched in mutation-intolerant genes and in regions under strong background selection. *Nat Genet*. 2018;50(3):381-389. doi:10.1038/s41588-018-0059-2

Selzam S, Ritchie SJ, Pingault J-B, Reynolds CA, O'Reilly PF, Plomin R. Comparing Within- and Between-Family Polygenic Score Prediction. *Am J Hum Genet*. 2019;105(2):351-363. doi:10.1016/j.ajhg.2019.06.006

Vilhjálmsson BJ, Yang J, Finucane HK, et al. Modeling Linkage Disequilibrium Increases Accuracy of Polygenic Risk Scores. *Am J Hum Genet*. 2015;97(4):576-592. doi:10.1016/j.ajhg.2015.09.001

Wray NR, Ripke S, Mattheisen M, et al. Genome-wide association analyses identify 44 risk variants and refine the genetic architecture of major depression. *Nat Genet*. 2018;50(5):668-681. doi:10.1038/s41588-018-0090-3

Supplementary Information 4. Multiple testing correction for regression analyses

The False Discovery Rate (FDR) method of correcting for multiple testing first requires the *P* values from the multiple tests to be ranked according to their significance levels. The FDR-adjusted *P* value is defined as the highest-ranking test for which the *P* value is less than or equal to the rank number divided by the total number of tests, multiplied by α (.05). The resulting value is referred to as corrected $q < .05$.

Linear regressions: Each subdomain at each age was first regressed separately on MDD GPS and schizophrenia GPS, at each GPS fraction (*f*) (1, 0.3, 0.01). Next, across GPS *f*, estimates for each subdomain at each age with the highest corresponding *z* statistic (i.e., the unstandardized estimate divided by its standard error) were selected. This group of estimates from the most predictive GPS *f* were then subjected to multiple testing correction. Of the 30 tests (5 subdomains, 3 ages, 2 GPS predictors), 13 were significant at $P < .05$. The resulting FDR-adjusted significance level was $P < .02$, at rank 12/30 (.016 < .020).

Multiple regressions: The most predictive GPS *f* (from the linear regressions) were entered into multiple-predictor (MDD GPS and schizophrenia GPS) regressions for each subdomain at each age separately and were subjected to multiple testing correction. Of the 30 tests (5 subdomains, 3 ages, 2 GPS predictors), 12 were significant at $P < .05$. The resulting FDR-adjusted significance level was $P < .015$, at rank 9/30 (.002 < .015).

Supplementary Information 5. Models used in confirmatory factor analysis

Model 1: A 1-factor model, representing total negative symptoms as a unidimensional construct. In this model, all indicators were specified to load onto a single factor. All parameters were freely estimated.

Model 2: A 2-factor model, representing the motivational-pleasure and expressive deficits as reflected in the current Diagnostic and Statistical Manual (DSM-5). In this model, items 1-4 were specified to load onto a factor reflecting an expressive deficit and items 5-8 were specified to load onto a factor reflecting a motivational-pleasure deficit. Factors were free to correlate and all parameters were freely estimated.

Model 3: A 4-factor model, derived through principal axis exploratory factor analysis with oblique rotation and using parallel analysis used for factor retention. Briefly, parallel analysis generates a random set of data with the same number of variables and observations as the empirical dataset and then calculates eigenvalues of the randomly generated correlation matrix. Eigenvalues of the correlation matrix in the empirical dataset that exceed those from the randomly generated data determine the number of factors to retain. For the models used in the confirmatory analyses, factors were determined by the items that had the highest loadings (no cross loadings were specified). At ages 16 and 17 (in both the main and pseudo-replications samples), items 1-2 were specified to load onto a factor reflecting flat affect, items 3-4 were specified to load onto a factor reflecting alogia, items 5-6 were specified to load onto a factor reflecting avolition and items 7-8 were specified to load onto a factor reflecting anhedonia-asociality. At age 22 in the main sample, item-to-factor configuration was the same except that item 7 was specified to load onto the factor reflecting avolition and item 8 was specified as a single-indicator factor reflecting asociality. In the pseudo-replication sample at age 22, the same model as at ages 16 and 17 was specified. Factors were free to correlate and all parameters were freely estimated, except at age 22 in the main sample where the factor loading for item 8 was fixed to 1 to achieve model identification and the residual variance fixed at 0.

Model 4: A 5-factor model, representing the subdomains highlighted in the National Institute of Mental Health (NIMH) consensus development conference. In this model, items 1-2 were specified to load onto a factor reflecting flat affect, items 3-4 were specified to load onto a factor reflecting alogia, items 5-6 were specified to load onto a factor reflecting avolition, item 7 was specified as a single-indicator factor reflecting anhedonia and item 8 was specified as a single-indicator factor reflecting asociality. The factor loadings for items 7 and 8 were fixed at 1 to achieve model identification and residual variances fixed at 0. Factors were free to correlate and all other parameters were freely estimated.

Model 5: A 5-factor hierarchical model, including the negative symptom dimensions reflected in the DSM-5 (motivational-pleasure and expressive deficits, as per Model 2) as second order factors, and the 5 NIMH subdomains (as per Model 4) as first order factors. In this model, influence on the items comes both directly from the first order factors and indirectly from the second order factors via the first order factors. The second order factors were free to correlate and the first order factors were uncorrelated.

Supplementary Information 6. Goodness-of-fit indices

The chi-square (χ^2) statistic (i.e., the *T* statistic, which is chi-square distributed) reflects the discrepancy between the model-implied variance-covariance matrix and the observed variance-covariance matrix. This is calculated as minus 2 times the difference in loglikelihood values between the test model and saturated model. Chi-square values were not used to assess fit given the tendency towards significance in large samples. The comparative fit index (CFI) compares the χ^2 and *df* of the test model with those of the baseline (means and variances only) model. The root mean square error of approximation (RMSEA) is a parsimony fit index, penalizing complex models. It assesses the extent to which the test model approximates the observed data taking into account *df* and sample size. The standardized root mean squared residual (SRMR) is an absolute fit index that averages the non-redundant residual correlations between the values implied by the test model and the observed data.

Supplementary Information 7. Measurement invariance

Testing for measurement invariance (MI) allows for the estimation of the extent to which a latent construct is the same between groups, or in the case of longitudinal modelling, over time. Increasing levels of measurement invariance are tested through a series of models. The first level in MI analysis is a test of configural invariance. This tests (only) whether the configuration of observed variables with their corresponding latent variables is the same across groups or at different time points. The next level is a test of metric invariance, often called 'weak' invariance. This tests whether factor loadings can be constrained to equality between groups or at different time-points. Essentially, this asks whether the latent variables explain a similar amount of the variance in each of the observed variables across the groups or at different time points. The next level is scalar (or 'strong') invariance, in which in addition to constraining factor loadings to equality, the intercepts of the observed variables are constrained to be equal. This asks the question whether, given the average score on each latent variable, the average score of each observed variable is the same or sufficiently similar between the groups. If scalar invariance is achieved, 'strict' invariance models can further be tested, though strict invariance is not usually necessary to conclude MI. One typical example of strict invariance is to additionally constrain residual variances to equality. If full MI is not achieved at any level, partial invariance models can be tested. This allows for the free estimation of the parameters for a particular item, usually guided by the modification indices and or theory.

Supplementary Table 1. The Twins Early Development Study (TEDS) Sample

	N parents contacted	*N* parents returned data	% return rate	*N* (approximate) not contacted from ONS sample owing to exclusions[1]
N parents that responded to initial ONS invitation, *N* = 16810 ('ONS sample')				
1st contact study[a]	16302	13488	82.74%	500
16-year study	10874	5123	47.11%	5900
17-year study	1773	1475	83.19%	See[2] below
21-year study	10451	5252	51.21%	6250

Note. ONS = Office for National Statistics. [a] Non-contact of families that responded to the initial ONS invitation for the 1st contact study was for several reasons: 1) Ambiguous or aesthetically spoiled ONS responses: Following contact of these families, some either changed their minds or withdrew from the study. 2) Responding to the ONS invitation too late. 3) Withdrawal from the study following initial acceptance. 4) Address and contact problems. [1] Exclusions were due to families withdrawing from the study, address problems, severe medical conditions, families being inactive, families with no recent data, and for 'other reasons', which are detailed in full on the TEDS data dictionary (https://www.teds.ac.uk/datadictionary/studies/returns/samples.htm). The TEDS data dictionary also lists the exact numbers of exclusions for each reason for each study. [2] The sample at 17 was a selected subset of 1773 of the families who had returned data at 16. Families were selected where at least 1 twin scored highly for at least 1 of the 6 subscales of the Specific Psychotic Experiences Questionnaire, or where neither twin scored highly for these measures.

Supplementary Table 2. Parent Data Returns at Ages 16, 17 and 22 for Main and Co-Twin Samples

	Main sample						Co-twin sample					
	Age 16 only	Age 17 only	Age 22 only	Age 16 and 17 only	Age 16 and 22 only	Age 16, 17 and 22	Age 16 only	Age 17 only	Age 22 only	Age 16 and 17 only	Age 16 and 22 only	Age 16, 17 and 22
N	857 (13.47%)	0 (0%)	1385 (21.77%)	260 (4.09%)	2648 (41.63%)	1211 (19.04%)	859 (13.49%)	0 (0%)	1388 (21.80%)	262 (4.12%)	2646 (41.56%)	1211 (19.02%)
SES	-0.18 (0.96)	NA	0.18 (1.02)	-0.23 (0.98)	0.37 (0.96)	0.28 (0.96)	-0.18 (0.96)	NA	0.18 (1.02)	-0.23 (0.98)	0.37 (0.96)	0.28 (0.96)
Female	45.97%	NA	50.40%	53.08%	57.02%	57.47%	49.24%	NA	50.36%	56.11%	55.78%	59.62%

Note: N = number of individuals for whom data was returned by their parents across data collection waves. SES = socio-economic status, reflecting a mean standardized SES composite score at first contact (SD in parentheses).

Supplementary Table 3. Descriptive Statistics for Negative Symptom Items, Subdomains and Totals at Ages 16, 17 and 22 in Main and Co-Twin Samples

	Main sample						Co-twin sample					
	16 years		17 years		22 years		16 years		17 years		22 years	
	Mean (SD)	Skewness	Mean (SD)	Skewness	Mean (SD)	Skewness	Mean (SD)	Skewness	Mean (SD)	Skewness	Mean (SD)	Skewness
Item 1	0.14 (0.44)	3.85	0.26 (0.59)	2.66	0.15 (0.46)	3.81	0.14 (0.44)	3.86	0.24 (0.57)	2.75	0.14 (0.56)	3.85
Item 2	0.16 (0.46)	3.33	0.25 (0.57)	2.63	0.26 (0.60)	2.72	0.16 (0.47)	3.47	0.23 (0.54)	2.71	0.25 (0.58)	2.73
Item 3	0.34 (0.65)	2.18	0.44 (0.72)	1.79	0.36 (0.70)	2.19	0.34 (0.67)	2.22	0.41 (0.70)	1.88	0.36 (0.70)	2.16
Item 4	0.35 (0.65)	2.07	0.42 (0.71)	1.81	0.39 (0.70)	1.96	0.35 (0.67)	2.15	0.37 (0.68)	1.95	0.39 (0.70)	1.96
Item 5	0.34 (0.65)	2.18	0.44 (0.74)	1.84	0.41 (0.71)	1.99	0.33 (0.65)	2.26	0.42 (0.70)	1.79	0.40 (0.71)	1.98
Item 6	0.35 (0.64)	2.04	0.43 (0.71)	1.76	0.42 (0.69)	1.82	0.33 (0.62)	2.11	0.43 (0.69)	1.70	0.41 (0.68)	1.86
Item 7	0.34 (0.65)	2.06	0.47 (0.76)	1.67	0.43 (0.73)	1.82	0.34 (0.65)	2.12	0.44 (0.72)	1.73	0.42 (0.70)	1.83
Item 8	0.19 (0.53)	3.31	0.29 (0.68)	2.61	0.25 (0.63)	2.86	0.18 (0.52)	3.37	0.27 (0.63)	2.63	0.24 (0.62)	2.91
Flat affect	0.15 (0.39)	3.65	0.26 (0.52)	2.67	0.20 (0.46)	3.10	0.15 (0.39)	3.57	0.24 (0.49)	2.66	0.20 (0.45)	3.04
Alogia	0.34 (0.60)	2.15	0.43 (0.67)	1.82	0.37 (0.63)	2.07	0.35 (0.62)	2.23	0.39 (0.64)	1.92	0.38 (0.63)	2.05
Avolition	0.34 (0.58)	2.20	0.44 (0.67)	1.87	0.41 (0.62)	1.90	0.33 (0.57)	2.21	0.43 (0.63)	1.77	0.41 (0.62)	1.87
Anhedonia	0.34 (0.65)	2.06	0.47 (0.76)	1.67	0.43 (0.73)	1.82	0.34 (0.65)	2.12	0.44 (0.72)	1.73	0.42 (0.70)	1.83
Asociality	0.19 (0.53)	3.31	0.29 (0.68)	2.61	0.25 (0.63)	2.86	0.18 (0.52)	3.37	0.27 (0.63)	2.63	0.24 (0.62)	2.91
Total NS	2.21 (3.21)	2.40	3.01 (4.07)	2.11	2.66 (3.64)	2.27	2.17 (3.18)	2.41	2.82 (3.81)	2.05	2.61 (3.57)	2.23
Coefficient α	0.83		0.88		0.83		0.83		0.87		0.83	

Note: *N* at age 16 in main sample = 4942-4971; *N* at age 17 in main sample = 1451-1469; *N* at age 22 in main sample = 5147-5177. *N* at age 16 in co-twin sample = 4945-4973; *N* at age 17 in co-twin sample = 1450-1473; *N* at age 22 in co-twin sample = 5154-5178. NS = negative symptoms. Flat affect is a composite of items 1 and 2, alogia is a composite of items 3 and 4, avolition is a composite of items 5 and 6, anhedonia is item 7 and asociality is item 8. Coefficient alpha (α) for items 1-8.

Supplementary Table 4. Proportion of Item-Level Data Present Across Ages for Main Sample

	PPBHS011	PPBHS021	PPBHS031	PPBHS041	PPBHS051
PPBHS011	0.784				
PPBHS021	0.782	0.783			
PPBHS031	0.782	0.782	0.783		
PPBHS041	0.781	0.780	0.781	0.782	
PPBHS051	0.781	0.780	0.780	0.779	0.782
PPBHS061	0.780	0.780	0.780	0.779	0.780
PPBHS071	0.779	0.778	0.779	0.778	0.779
PPBHS081	0.780	0.779	0.779	0.779	0.779
PPL2S011	0.231	0.231	0.231	0.231	0.230
PPL2S021	0.231	0.230	0.230	0.230	0.230
PPL2S031	0.231	0.230	0.230	0.230	0.230
PPL2S041	0.231	0.230	0.230	0.230	0.230
PPL2S051	0.231	0.231	0.231	0.231	0.230
PPL2S061	0.231	0.230	0.230	0.230	0.230
PPL2S071	0.230	0.230	0.229	0.229	0.229
PPL2S081	0.229	0.229	0.229	0.229	0.228
U1PS091	0.600	0.599	0.600	0.599	0.600
U1PS051	0.601	0.600	0.600	0.599	0.600
U1PS031	0.602	0.600	0.601	0.600	0.601
U1PS021	0.602	0.600	0.601	0.600	0.601
U1PS061	0.599	0.598	0.599	0.598	0.598
U1PS011	0.601	0.600	0.601	0.600	0.600
U1PS071	0.601	0.600	0.600	0.599	0.600
U1PS101	0.601	0.600	0.601	0.600	0.600

	PPBHS061	PPBHS071	PPBHS081	PPL2S011	PPL2S021
PPBHS061	0.782				
PPBHS071	0.779	0.781			
PPBHS081	0.779	0.778	0.782		
PPL2S011	0.230	0.230	0.230	0.231	
PPL2S021	0.230	0.230	0.230	0.230	0.231
PPL2S031	0.230	0.229	0.230	0.230	0.229
PPL2S041	0.230	0.230	0.230	0.230	0.230
PPL2S051	0.230	0.230	0.230	0.231	0.230
PPL2S061	0.230	0.230	0.230	0.230	0.230
PPL2S071	0.229	0.229	0.229	0.229	0.229
PPL2S081	0.228	0.228	0.228	0.228	0.228
U1PS091	0.598	0.598	0.598	0.187	0.187
U1PS051	0.599	0.598	0.599	0.188	0.188
U1PS031	0.599	0.599	0.600	0.188	0.188
U1PS021	0.599	0.599	0.600	0.188	0.187
U1PS061	0.597	0.597	0.597	0.188	0.187
U1PS011	0.599	0.599	0.599	0.188	0.188
U1PS071	0.599	0.598	0.599	0.188	0.187
U1PS101	0.599	0.599	0.599	0.188	0.188

	PPL2S031	PPL2S041	PPL2S051	PPL2S061	PPL2S071
PPL2S031	0.231				
PPL2S041	0.230	0.231			
PPL2S051	0.230	0.230	0.231		
PPL2S061	0.230	0.230	0.230	0.231	
PPL2S071	0.229	0.229	0.229	0.229	0.230

PPL2S081	0.228	0.228	0.228	0.229	0.227
U1PS091	0.187	0.187	0.188	0.188	0.186
U1PS051	0.188	0.188	0.188	0.188	0.187
U1PS031	0.188	0.188	0.188	0.188	0.187
U1PS021	0.188	0.188	0.188	0.188	0.187
U1PS061	0.187	0.187	0.188	0.188	0.187
U1PS011	0.188	0.188	0.188	0.188	0.187
U1PS071	0.187	0.187	0.188	0.188	0.187
U1PS101	0.188	0.188	0.188	0.188	0.187

	PPL2S081	U1PS091	U1PS051	U1PS031	U1PS021
PPL2S081	0.229				
U1PS091	0.186	0.815			
U1PS051	0.187	0.812	0.815		
U1PS031	0.187	0.813	0.814	0.816	
U1PS021	0.186	0.812	0.813	0.814	0.815
U1PS061	0.186	0.811	0.811	0.811	0.811
U1PS011	0.187	0.813	0.813	0.814	0.813
U1PS071	0.186	0.812	0.812	0.813	0.812
U1PS101	0.187	0.812	0.812	0.813	0.812

	U1PS061	U1PS011	U1PS071	U1PS101
U1PS061	0.813			
U1PS011	0.811	0.816		
U1PS071	0.810	0.813	0.815	
U1PS101	0.810	0.812	0.812	0.815

Note: Variable names relate to negative symptom items 01-08, with prefixes ‘PPBHS’ for age 16, ‘PPL2S’ for age 17, and ‘UP1PS’ for age 22. The ‘1’ following the item numbers refers to twin 1 (randomly assigned). Values represent the proportion of individuals contributing data to the variance and covariance calculations. For example, the value for PPBHS011 and PPL2S011 reflects that 23.1% of individuals contributed data to the covariance calculation for item 1 at age 16 and item 1 at age 17. Note that for the age 22 variables; item 01 is labelled as item 09, item 02 is labelled as item 05, item 03 is labelled as item 03, item 04 is labelled as item 02, item 05 is labelled as item 06, item 06 is labelled as item 01, item 07 is labelled as item 07, and item 08 is labelled as item 10.

Supplementary Table 5. Confirmatory Factor Analysis of Negative Symptoms at Ages 16, 17 and 22 in Co-Twin Sample: Model Fit Results

	Parameters	Log-likelihood	AIC	BIC	χ^2 value (*df*)	CFI	RMSEA [90% CI]	SRMR
16 years								
1-factor model	24	-29,108.55	58,265.11	58,421.41	1,219.01 (20), *P*<.001	0.79	0.17 [0.16, 0.18]	0.07
2-factor model	25	-28,159.43	56,368.86	56,531.68	404.88 (19), *P*<.001	0.93	0.10 [0.09, 0.11]	0.05
4-factor model	30	-27,736.60	55,533.20	55,728.58	76.58 (14), *P*<.001	0.99	0.05 [0.04, 0.06]	0.02
**5-factor model**	**32**	**-27,694.95**	**55,453.91**	**55,662.31**	**42.99 (12), *P*<.001**	**0.99**	**0.04 [0.02, 0.05]**	**0.01**
5H-factor model	28	-27,766.26	55,588.53	55,770.88	99.75 (16), *P*<.001	0.99	0.05 [0.04, 0.06]	0.02
17 years								
1-factor model	24	-9,373.98	18,795.97	18,923.05	393.06 (20), *P*<.001	0.85	0.16 [0.15, 0.18]	0.06
2-factor model	25	-9,099.30	18,248.61	18,380.99	126.87 (19), *P*<.001	0.96	0.09 [0.07, 0.10]	0.04
4-factor model	29	-9,000.98	18,061.96	18,220.82	30.80 (14), *P*=.006	0.99	0.04 [0.02, 0.06]	0.02
**5-factor model**	**32**	**-8,988.13**	**18,040.26**	**18,209.70**	**17.69 (12), *P*=.13**	**1.00**	**0.02 [0.00, 0.05]**	**0.01**
5H-factor model	28	-9,008.63	18,073.26	18,221.53	37.31 (16), *P*=.002	0.99	0.04 [0.03, 0.06]	0.02
22 years								
1-factor model	24	-34,117.00	68,282.00	68,439.26	927.70 (20), *P*<.001	0.86	0.14 [0.13, 0.15]	0.06
2-factor model	25	-33,667.27	67,384.54	67,548.36	519.30 (19), *P*<.001	0.92	0.11 [0.10, 0.11]	0.05
4-factor model	29	-33,398.28	66,854.57	67,044.60	285.64 (15), *P*<.001	0.96	0.08 [0.08, 0.09]	0.03
**5-factor model**	**32**	**-33,271.48**	**66,606.95**	**66,816.64**	**161.11 (12), *P*<.001**	**0.98**	**0.07 [0.06, 0.08]**	**0.02**
5H-factor model	28	-33,358.65	66,773.30	66,956.78	242.01 (16), *P*<.001	0.97	0.08 [0.07, 0.08]	0.03

Note: *N* at age 16 = 4977; *N* at age 17 = 1743; *N* at age 22 = 5179. Robust maximum likelihood estimation (MLR). 5H-factor model = 5-factor hierarchical model. AIC = Akaike's Information Criterion. BIC = Bayesian Information Criterion. χ^2 = chi-square value. CFI = comparative fit index. RMSEA = root mean square error of approximation. SRMR = standardized root mean square residual. CI = confidence intervals. Chi-square for baseline models: At 16, χ^2 (28) = 4995.48, *P*< .001. At 17, χ^2 (28) = 2382.13, *P*< .001. At 22, χ^2 (28) = 5995.12, *P*< .001. Bold typeset represents best fitting model at each age.

Supplementary Table 6. Measurement Invariance of the 5-Factor Structure of Negative Symptoms at Age 16 between the Main and Co-Twin Samples: Goodness-of-Fit Indices

	Parameters	Fit indices			Comparison of fit indices between nested models		
		CFI	RMSEA [90% CI]	SRMR	Δ CFI	Δ RMSEA	Δ SRMR
Configural invariance model (no constraints)	64	0.996	0.031 [0.024, 0.040]	0.011	-	-	-
Metric invariance model (factor loadings constrained)	58	0.996	0.026 [0.018, 0.035]	0.014	0.000	0.005	-0.003
Scalar invariance model (factor loadings and intercepts constrained)	55	0.996	0.025 [0.017, 0.033]	0.014	0.000	0.001	0.000
Strict invariance model (factor loadings, intercepts and residual variances constrained)	49	0.997	0.022 [0.013, 0.029]	0.015	-0.001	0.003	-0.001

Note: N = 9951 (main sample N = 4974, co-twin sample N = 4977). Cluster-robust SE. Robust maximum likelihood estimation (MLR). CFI = comparative fit index. RMSEA = root mean square error of approximation. SRMR = standardized root mean square residual. CI = confidence intervals. Δ denotes change value. Chi-square for baseline model: χ^2 (56) = 10599.18, $P <$ .001.

Supplementary Table 7. Measurement Invariance of the 5-Factor Structure of Negative Symptoms at Age 17 between the Main and Co-Twin Samples: Goodness-of-Fit Indices

	Parameters	Fit indices			Comparison of fit indices between nested models		
		CFI	RMSEA [90% CI]	SRMR	Δ CFI	Δ RMSEA	Δ SRMR
Configural invariance model (no constraints)	64	1.000	0.010 [0.000, 0.031]	0.010	-	-	-
Metric invariance model (factor loadings constrained)	58	1.000	0.000 [0.000, 0.025]	0.030	0.000	0.010	0.020
Scalar invariance model (factor loadings and intercepts constrained)	55	1.000	0.000 [0.000, 0.023]	0.030	0.000	0.000	0.000
Strict invariance model (factor loadings, intercepts and residual variances constrained)	49	1.000	0.000 [0.000, 0.020]	0.033	0.000	0.000	-0.003

Note: N = 2942 (main sample N = 1469, co-twin sample N = 1473). Cluster-robust SE. Robust maximum likelihood estimation (MLR). CFI = comparative fit index. RMSEA = root mean square error of approximation. SRMR = standardized root mean square residual. CI = confidence intervals. Δ denotes change value. Chi-square for baseline model: χ^2 (56) = 5018.35, $P <$ .001.

Supplementary Table 8. Measurement Invariance of the 5-Factor Structure of Negative Symptoms at Age 22 between the Main and Co-Twin Samples: Goodness-of-Fit Indices

	Parameters	Fit indices			Comparison of fit indices between nested models		
		CFI	RMSEA [90% CI]	SRMR	Δ CFI	Δ RMSEA	Δ SRMR
Configural invariance model (no constraints)	64	0.982	0.064 [0.057, 0.071]	0.021	-	-	-
Metric invariance model (factor loadings constrained)	58	0.982	0.056 [0.050, 0.063]	0.021	0.000	0.008	0.000
Scalar invariance model (factor loadings and intercepts constrained)	55	0.982	0.053 [0.048, 0.060]	0.021	0.000	0.003	0.000
Strict invariance model (factor loadings, intercepts and residual variances constrained)	49	0.983	0.048 [0.043, 0.054]	0.022	-0.001	0.005	-0.001

Note: N = 10149 (main sample N = 5179, co-twin sample N = 5181). Cluster-robust SE. Robust maximum likelihood estimation (MLR). CFI = comparative fit index. RMSEA = root mean square error of approximation. SRMR = standardized root mean square residual. CI = confidence intervals. Δ denotes change value. Chi-square for baseline model: χ^2 (56) = 12155.66, $P<$ .001.

Supplementary Table 9. Confirmatory Factor Analysis of Negative Symptoms at Ages 16, 17 and 22 in Main Sample using Diagonally Weighted Least Squares Estimation for Categorical Indicators: Model Fit Results

	Parameters	χ^2 value (*df*)	CFI	RMSEA [90% CI]	SRMR
16 years					
1-factor model	32	1962.14 (20), *P*<.001	0.92	0.14 [0.14, 0.15]	0.08
2-factor model	33	587.79 (19), *P*<.001	0.98	0.08 [0.07, 0.08]	0.05
4-factor model	38	177.28 (14), *P*<.001	0.99	0.05 [0.04, 0.05]	0.03
**5-factor model**	**40**	**39.48 (12), *P*<.001**	**0.99**	**0.02 [0.01, 0.03]**	**0.01**
5H-factor model	35	231.64 (17), *P*<.001	0.99	0.05 [0.05, 0.06]	0.03
17 years					
1-factor model	32	640.26 (20), *P*<.001	0.95	0.15 [0.14, 0.16]	0.07
2-factor model	33	194.54 (19), *P*<.001	0.99	0.08 [0.07, 0.09]	0.04
4-factor model	38	40.86 (14), *P*<.001	0.99	0.04 [0.02, 0.05]	0.02
**5-factor model**	**40**	**17.66 (12), *P*=.13**	**0.99**	**0.02 [0.00, 0.04]**	**0.01**
5H-factor model	35	67.18 (17), *P*<.001	0.99	0.05 [0.03, 0.06]	0.02
22 years					
1-factor model	32	1515.33 (20), *P*<.001	0.93	0.12 [0.12, 0.13]	0.06
2-factor model	33	643.38 (19), *P*<.001	0.92	0.08 [0.07, 0.09]	0.04
4-factor model	38	251.79 (14), *P*<.001	0.99	0.06 [0.05, 0.06]	0.03
**5-factor model**	**40**	**145.94 (12), *P*<.001**	**0.99**	**0.05 [0.04, 0.05]**	**0.02**
5H-factor model	35	220.84 (17), *P*<.001	0.99	0.05 [0.04, 0.05]	0.03

Note: *N* at age 16 = 4974; *N* at age 17 = 1469; *N* at age 22 = 5179. Diagonally weighted least squares estimation with robust standard errors (WLSMV), using pair-wise present data. 5H-factor model = 5-factor hierarchical model. χ^2 = chi-square value. CFI = comparative fit index. RMSEA = root mean square error of approximation. SRMR = standardized root mean square residual. CI = confidence intervals. Chi-square for baseline models: At 16, χ^2 (28) = 23870.96, *P*< .001. At 17, χ^2 (28) = 12155.78, *P*< .001. At 22, χ^2 (28) = 21938.18, *P*< .001. Bold typeset represents best fitting model at each age.

Supplementary Table 10. Parameter Estimates from the 5-Factor Model of Negative Symptoms at Age 16 in Main Sample

	Estimate	SE	*z*	*P*	Fully standardized path coefficient
Factor loadings					
**Flat affect**					
Item 1	0.28	0.02	17.19	<.001	0.63
Item 2	0.39	0.02	23.77	<.001	0.83
**Alogia**					
Item 3	0.55	0.02	35.18	<.001	0.83
Item 4	0.55	0.02	36.16	<.001	0.84
**Avolition**					
Item 5	0.47	0.02	29.87	<.001	0.72
Item 6	0.55	0.02	36.06	<.001	0.86
**Anhedonia**					
Item 7	1.00[a]	-	-	-	1.00
**Asociality**					
Item 8	1.00[a]	-	-	-	1.00
Covariances					
**Flat affect**					
Alogia	0.71	0.02	29.87	<.001	0.71
Avolition	0.56	0.03	21.57	<.001	0.56
Anhedonia	0.28	0.02	15.71	<.001	0.44
Asociality	0.23	0.02	12.34	<.001	0.42
**Alogia**					
Avolition	0.54	0.02	25.61	<.001	0.54
Anhedonia	0.27	0.02	17.33	<.001	0.41
Asociality	0.18	0.02	12.12	<.001	0.33
**Avolition**					
Anhedonia	0.43	0.02	27.20	<.001	0.66
Asociality	0.19	0.01	12.93	<.001	0.35
**Anhedonia**					
Asociality	0.15	0.01	13.69	<.001	0.42
Intercepts					
Item 1	0.14	0.01	22.18	<.001	0.31
Item 2	0.16	0.01	24.82	<.001	0.35
Item 3	0.34	0.01	36.58	<.001	0.52
Item 4	0.35	0.01	37.85	<.001	0.54
Item 5	0.34	0.01	36.43	<.001	0.52

Item 6	0.35	0.01	39.12	<.001	0.56
Item 7	0.35	0.01	37.45	<.001	0.53
Item 8	0.19	0.01	24.86	<.001	0.35
Variances					
Item 1	0.12	0.01	16.72	<.001	0.60
Item 2	0.07	0.01	10.51	<.001	0.31
Item 3	0.13	0.01	13.34	<.001	0.30
Item 4	0.13	0.01	13.74	<.001	0.29
Item 5	0.20	0.01	21.82	<.001	0.48
Item 6	0.10	0.01	11.44	<.001	0.26
Item 7	0.00[a]	-	-	-	0.00
Item 8	0.00[a]	-	-	-	0.00
**Flat affect**	1.00[b]	-	-	-	1.00
**Alogia**	1.00[b]	-	-	-	1.00
**Avolition**	1.00[b]	-	-	-	1.00
**Anhedonia**	0.42	0.02	28.17	<.001	1.00
**Asociality**	0.29	0.02	19.03	<.001	1.00

Note: N = 4974. Estimate = unstandardized factor loading. [a] = Factor loadings fixed to 1 and residual variances fixed to 0 for factors with single indicators. Anhedonia and asociality have freely estimated variances due to the fixed factor loadings. [b] = Factor variances fixed to 1 for factor scaling.

Supplementary Table 11. Parameter Estimates from the 5-Factor Model of Negative Symptoms at Age 17 in Main Sample

	Estimate	SE	*z*	*P*	Fully standardized path coefficient
Factor loadings					
**Flat affect**					
Item 1	0.39	0.03	14.04	<.001	0.65
Item 2	0.49	0.03	17.07	<.001	0.85
**Alogia**					
Item 3	0.61	0.03	24.95	<.001	0.86
Item 4	0.63	0.03	25.09	<.001	0.88
**Avolition**					
Item 5	0.57	0.03	19.94	<.001	0.77
Item 6	0.63	0.03	25.49	<.001	0.89
**Anhedonia**					
Item 7	1.00[a]	-	-	-	1.00
**Asociality**					
Item 8	1.00[a]	-	-	-	1.00
Covariances					
**Flat affect**					
Alogia	0.78	0.03	23.29	<.001	0.78
Avolition	0.67	0.04	19.16	<.001	0.67
Anhedonia	0.42	0.03	13.82	<.001	0.56
Asociality	0.32	0.03	9.83	<.001	0.47
**Alogia**					
Avolition	0.64	0.03	21.25	<.001	0.64
Anhedonia	0.41	0.03	14.61	<.001	0.54
Asociality	0.32	0.03	10.21	<.001	0.47
**Avolition**					
Anhedonia	0.55	0.03	20.43	<.001	0.73
Asociality	0.36	0.03	11.47	<.001	0.53
**Anhedonia**					
Asociality	0.27	0.03	10.58	<.001	0.52
Intercepts					
Item 1	0.26	0.02	16.75	<.001	0.44
Item 2	0.25	0.02	16.82	<.001	0.44
Item 3	0.44	0.02	23.32	<.001	0.61
Item 4	0.42	0.02	22.42	<.001	0.59
Item 5	0.44	0.02	22.64	<.001	0.59
Item 6	0.43	0.02	23.35	<.001	0.61

Item 7	0.47	0.02	23.80	<.001	0.62
Item 8	0.29	0.02	16.49	<.001	0.43
Variances					
Item 1	0.21	0.02	10.22	<.001	0.58
Item 2	0.09	0.01	6.94	<.001	0.27
Item 3	0.14	0.02	8.64	<.001	0.26
Item 4	0.12	0.01	8.44	<.001	0.23
Item 5	0.23	0.02	12.56	<.001	0.41
Item 6	0.11	0.01	7.50	<.001	0.22
Item 7	0.00[a]	-	-	-	0.00
Item 8	0.00[a]	-	-	-	0.00
**Flat affect**	1.00[b]	-	-	-	1.00
**Alogia**	1.00[b]	-	-	-	1.00
**Avolition**	1.00[b]	-	-	-	1.00
**Anhedonia**	0.58	0.03	18.49	<.001	1.00
**Asociality**	0.46	0.04	13.02	<.001	1.00

Note: *N* = 1469.Estimate = unstandardized factor loading. [a] = Factor loadings fixed to 1 and residual variances fixed to 0 for factors with single indicators. Anhedonia and asociality have freely estimated variances due to the fixed factor loadings. [b] = Factor variances fixed to 1 for factor scaling.

Supplementary Table 12. Parameter Estimates from the 5-Factor Model of Negative Symptoms at Age 22 in Main Sample

	Estimate	SE	*z*	*P*	Fully standardized path coefficient
Factor loadings					
**Flat affect**					
Item 1	0.28	0.02	17.96	<.001	0.61
Item 2	0.46	0.02	26.86	<.001	0.76
**Alogia**					
Item 3	0.56	0.02	36.05	<.001	0.80
Item 4	0.57	0.02	37.67	<.001	0.81
**Avolition**					
Item 5	0.53	0.02	33.31	<.001	0.74
Item 6	0.52	0.02	35.14	<.001	0.75
**Anhedonia**					
Item 7	1.00[a]	-	-	-	1.00
**Asociality**					
Item 8	1.00[a]	-	-	-	1.00
Covariances					
**Flat affect**					
Alogia	0.82	0.02	40.22	<.001	0.82
Avolition	0.71	0.02	29.26	<.001	0.71
Anhedonia	0.38	0.02	19.95	<.001	0.52
Asociality	0.36	0.02	17.52	<.001	0.57
**Alogia**					
Avolition	0.60	0.02	28.22	<.001	0.60
Anhedonia	0.32	0.02	19.49	<.001	0.43
Asociality	0.28	0.02	17.21	<.001	0.45
**Avolition**					
Anhedonia	0.46	0.02	28.51	<.001	0.63
Asociality	0.30	0.02	18.03	<.001	0.48
**Anhedonia**					
Asociality	0.21	0.01	16.46	<.001	0.47
Intercepts					
Item 1	0.15	0.01	22.65	<.001	0.32
Item 2	0.26	0.01	30.82	<.001	0.43
Item 3	0.36	0.01	36.92	<.001	0.51
Item 4	0.39	0.01	40.15	<.001	0.56
Item 5	0.41	0.01	40.98	<.001	0.57
Item 6	0.42	0.01	43.28	<.001	0.60

Item 7	0.43	0.01	42.88	<.001	0.60
Item 8	0.25	0.01	28.99	<.001	0.40
Variances					
Item 1	0.14	0.01	17.18	<.001	0.63
Item 2	0.15	0.01	15.16	<.001	0.42
Item 3	0.18	0.01	15.45	<.001	0.36
Item 4	0.17	0.01	15.82	<.001	0.34
Item 5	0.23	0.01	20.08	<.001	0.45
Item 6	0.21	0.01	18.86	<.001	0.43
Item 7	0.00[a]	-	-	-	0.00
Item 8	0.00[a]	-	-	-	0.00
**Flat affect**	1.00[b]	-	-	-	1.00
**Alogia**	1.00[b]	-	-	-	1.00
**Avolition**	1.00[b]	-	-	-	1.00
**Anhedonia**	0.53	0.02	31.98	<.001	1.00
**Asociality**	0.39	0.02	22.45	<.001	1.00

Note: N = 5179. Estimate = unstandardized factor loading. [a] = Factor loadings fixed to 1 and residual variances fixed to 0 for factors with single indicators. Anhedonia and asociality have freely estimated variances due to the fixed factor loadings. [b] = Factor variances fixed to 1 for factor scaling.

Supplementary Table 13. Communality and Uniqueness Estimates from the 5-factor Model of Negative Symptoms at Ages 16, 17 and 22 in Main Sample

	Factor	Pattern coefficient	Communality	Uniqueness
16 years				
Item 1	Flat affect	0.63	0.40	0.60
Item 2	Flat affect	0.83	0.69	0.31
Item 3	Alogia	0.83	0.69	0.31
Item 4	Alogia	0.84	0.71	0.29
Item 5	Avolition	0.72	0.52	0.48
Item 6	Avolition	0.86	0.74	0.26
Item 7	Anhedonia	1.00	1.00	0.00
Item 8	Asociality	1.00	1.00	0.00
17 years				
Item 1	Flat affect	0.65	0.42	0.58
Item 2	Flat affect	0.85	0.72	0.27
Item 3	Alogia	0.86	0.74	0.26
Item 4	Alogia	0.88	0.77	0.23
Item 5	Avolition	0.77	0.59	0.41
Item 6	Avolition	0.89	0.79	0.22
Item 7	Anhedonia	1.00	1.00	0.00
Item 8	Asociality	1.00	1.00	0.00
22 years				
Item 1	Flat affect	0.61	0.37	0.63
Item 2	Flat affect	0.76	0.58	0.42
Item 3	Alogia	0.80	0.64	0.36
Item 4	Alogia	0.81	0.66	0.34
Item 5	Avolition	0.74	0.55	0.45
Item 6	Avolition	0.75	0.56	0.44
Item 7	Anhedonia	1.00	1.00	0.00
Item 8	Asociality	1.00	1.00	0.00

Note: Pattern coefficient = correlation between factor and item (fully standardized path coefficient). Communality = squared pattern coefficient (i.e., a^2), percentage of variance in item explained by the factor. Uniqueness = residual variance (i.e., 1 - a^2), percentage of variance not explained by the factor. Item 7 and item 8 directional paths fixed to 1 and residual variances fixed to 0. Total variance in the items explained by the factors is the sum of the estimated squared pattern coefficients divided by the number of items. Total variance explained at 16 = 3.75/6, 62.50%; at 17 = 4.03/6, 67.16.%; at 22 = 3.36/6, 56%.

Supplementary Table 14. Longitudinal Measurement Invariance of the 5-Factor Structure of Negative Symptoms between Ages 16, 17 and 22 in the Main Sample: Goodness-of-Fit Indices

	Parameters	Fit indices			Comparison of fit indices between nested models		
		CFI	RMSEA [90% CI]	SRMR	Δ CFI	Δ RMSEA	Δ SRMR
Configural invariance model (no constraints)	189	0.992	0.014 [0.012, 0.016]	0.016	-	-	-
Metric invariance model (factor loadings constrained)	183	0.991	0.014 [0.012, 0.016]	0.018	0.001	0.000	0.002
Scalar invariance model (factor loadings and intercepts constrained)	177	0.988	0.016 [0.014, 0.018]	0.020	0.003	-0.002	-0.002
Strict invariance model (factor loadings, intercepts and residual variances constrained)	165	0.968	0.025 [0.023, 0.027]	0.030	0.020	-0.009	-0.010
Partial strict invariance model (factor loadings, intercepts and residual variances constrained, excluding item 2)[a]	171	0.980	0.020 [0.019, 0.022]	0.026	0.008[b]	-0.004[b]	-0.006[b]

Note: N = 6330. Robust maximum likelihood estimation (MLR). CFI = comparative fit index. RMSEA = root mean square error of approximation. SRMR = standardized root mean square residual. CI = confidence intervals. Δ denotes change value. [a]The change in CFI value from the scalar model to the strict model exceeded the acceptable limit (of 0.010). Consultation of the modification indices and subsequent free estimation of the item 2 parameters provided acceptable deterioration in model fit. [b]Change values compared to scalar invariance model. Chi-square for baseline model: χ^2 (276) = 20334, $P < .001$.

Supplementary Table 15. Longitudinal Measurement Invariance of the 5-Factor Structure of Negative Symptoms between Ages 16, 17 and 22 in the Co-Twin Sample: Goodness-of-Fit Indices

	Parameters	Fit indices			Comparison of fit indices between nested models		
		CFI	RMSEA [90% CI]	SRMR	Δ CFI	Δ RMSEA	Δ SRMR
Configural invariance model (no constraints)	189	0.986	0.017 [0.015, 0.019]	0.018	-	-	-
Metric invariance model (factor loadings constrained)	183	0.984	0.018 [0.016, 0.020]	0.021	0.002	-0.001	-0.003
Scalar invariance model (factor loadings and intercepts constrained)	177	0.981	0.019 [0.017, 0.021]	0.022	0.003	-0.001	-0.001
Strict invariance model (factor loadings, intercepts and residual variances constrained)	165	0.967	0.025 [0.023, 0.027]	0.030	0.014	-0.006	-0.008
Partial strict invariance model (factor loadings, intercepts and residual variances constrained, excluding item 2)[a]	171	0.976	0.022 [0.020, 0.023]	0.025	0.005[b]	-0.003[b]	-0.003[b]

Note: $N = 6336$. Robust maximum likelihood estimation (MLR). CFI = comparative fit index. RMSEA = root mean square error of approximation. SRMR = standardized root mean square residual. CI = confidence intervals. Δ denotes change value. [a]The change in CFI value from the scalar model to the strict model exceeded the acceptable limit (of 0.010). Consultation of the modification indices and subsequent free estimation of the item 2 parameters provided acceptable deterioration in model fit. [b]Change values compared to scalar invariance model. Chi-square for baseline model: χ^2 (276) = 19069.11, $P < .001$.

Supplementary Table 16. Linear Regression Results for Subdomain Mean Scores Regressed on Major Depressive Disorder GPS

	Age 16		Age 17		Age 22	
	b (SE)	*z (P)*	*b* (SE)	*z (P)*	*b* (SE)	*z (P)*
Flat affect						
GPS *f*						
1	0.019 (0.005)	3.465 (.001)	0.016 (0.012)	1.387 (.165)	0.018 (0.006)	3.191 (.001)
0.3	0.019 (0.005)	**<u>3.504 (<.001)</u>**	0.016 (0.012)	<u>1.410 (.159)</u>	0.018 (0.006)	**<u>3.195 (.001)</u>**
0.01	0.011 (0.005)	1.981 (.048)	0.002 (0.012)	0.148 (.882)	0.006 (0.006)	0.978 (.328)
Alogia						
GPS *f*						
1	0.010 (0.008)	<u>1.289 (.197)</u>	0.019 (0.015)	<u>1.253 (.210)</u>	0.009 (0.008)	<u>1.072 (.284)</u>
0.3	0.010 (0.008)	1.252 (.211)	0.019 (0.015)	1.244 (.213)	0.009 (0.008)	1.062 (.288)
0.01	-0.002 (0.008)	-0.304 (.761)	0.010 (0.015)	0.688 (.492)	0.004 (0.008)	0.516 (.606)
Avolition						
GPS *f*						
1	0.030 (0.007)	3.978 (<.001)	0.054 (0.016)	**<u>3.367 (.001)</u>**	0.028 (0.008)	**<u>3.487 (<.001)</u>**
0.3	0.030 (0.007)	**<u>3.986 (<.001)</u>**	0.053 (0.016)	3.351 (.001)	0.028 (0.008)	3.461 (.001)
0.01	0.021 (0.008)	2.612 (.009)	0.029 (0.017)	1.736 (.082)	0.007 (0.008)	0.893 (.372)
Anhedonia						
GPS *f*						
1	0.029 (0.009)	**<u>3.359 (.001)</u>**	0.046 (0.019)	**<u>2.433 (.015)</u>**	0.030 (0.009)	**<u>3.233 (.001)</u>**
0.3	0.029 (0.009)	3.348 (.001)	0.046 (0.019)	2.399 (.016)	0.030 (0.009)	3.200 (.001)
0.01	0.024 (0.009)	2.671 (.008)	0.010 (0.019)	0.538 (.591)	0.019 (0.009)	1.986 (.047)
Asociality						
GPS *f*						
1	0.028 (0.006)	**<u>4.321 (<.001)</u>**	0.025 (0.016)	<u>1.539 (.124)</u>	0.029 (0.008)	3.693 (<.001)
0.3	0.028 (0.006)	4.270 (<.001)	0.024 (0.016)	1.462 (.144)	0.029 (0.008)	**<u>3.702 (<.001)</u>**
0.01	0.010 (0.007)	1.326 (.185)	-0.008 (0.018)	-0.440 (.660)	0.014 (0.008)	1.702 (.089)

Note: N age 16= 5971-6006. *N* age 17 = 1791-1818. *N* age 22 = 6259-6278. Subdomain mean scores regressed on schizophrenia GPS. Related and unrelated individuals included, using cluster-robust SE. GPS = genome-wide polygenic score. *f* = fraction of causal markers. *b* = unstandardized regression coefficient. Underlined typeset represents most predictive *f* for each subdomain at each age. Bold typeset represents significance under corrected *q* <.05 threshold (FDR-adjusted *P* <.020).

Supplementary Table 17. Linear Regression Results for Subdomain Mean Scores Regressed on Schizophrenia GPS

	Age 16		Age 17		Age 22	
	b (SE)	*z (P)*	*b* (SE)	*z (P)*	*b* (SE)	*z (P)*
Flat affect						
GPS *f*						
1	0.014 (0.005)	**<u>2.659 (.008)</u>**	0.027 (0.012)	<u>2.162 (.031)</u>	0.001 (0.006)	0.147 (.883)
0.3	0.013 (0.005)	2.467 (.014)	0.023 (0.012)	1.848 (.065)	0.003 (0.006)	0.488 (.626)
0.01	-0.003 (0.006)	-0.525 (.600)	0.002 (0.0212)	0.192 (.848)	-0.004 (0.006)	<u>-0.737 (.461)</u>
Alogia						
GPS *f*						
1	0.002 (0.007)	0.307 (.759)	0.006 (0.016)	<u>0.387 (.699)</u>	-0.001 (0.008)	-0.032 (.974)
0.3	0.007 (0.008)	<u>0.893 (.372)</u>	0.003 (0.015)	0.182 (.855)	-0.000 (0.008)	-0.004 (.997)
0.01	-0.002 (0.008)	-0.304 (.761)	-0.001 (0.015)	-0.069 (.945)	0.002 (0.008)	<u>0.225 (.822)</u>
Avolition						
GPS *f*						
1	0.001 (0.008)	0.108 (.914)	0.038 (0.016)	**<u>2.404 (.016)</u>**	0.003 (0.008)	0.345 (.730)
0.3	0.001 (0.008)	0.135 (.892)	0.031 (0.016)	2.021 (.043)	-0.001 (0.008)	-0.090 (.928)
0.01	-0.004 (0.008)	<u>-0.496 (.620)</u>	0.008 (0.016)	0.478 (.632)	-0.006 (0.008)	<u>-0.738 (.460)</u>
Anhedonia						
GPS *f*						
1	0.005 (0.009)	0.534 (.593)	0.014 (0.018)	0.777 (.437)	-0.006 (0.010)	-0.639 (.523)
0.3	0.008 (0.009)	<u>0.922 (.356)</u>	0.017 (0.017)	<u>0.967 (.334)</u>	-0.007 (0.009)	<u>-0.729 (.466)</u>
0.01	-0.002 (0.009)	-0.268 (.789)	-0.007 (0.018)	-0.366 (.714)	-0.003 (0.009)	-0.310 (.757)
Asociality						
GPS *f*						
1	0.004 (0.007)	0.511 (.609)	0.005 (0.016)	0.312 (.755)	0.007 (0.008)	<u>0.893 (.372)</u>
0.3	0.001 (0.007)	0.133 (.894)	0.001 (0.015)	0.043 (.966)	0.005 (0.007)	0.652 (.515)
0.01	-0.007 (0.008)	<u>-0.858 (.391)</u>	-0.019 (0.016)	<u>-1.153 (.249)</u>	-0.004 (0.008)	-0.532 (.595)

Note: N age 16 = 5971-6006. *N* age 17 = 1791-1818. *N* age 22 = 6259-6278. Subdomain mean scores regressed on schizophrenia GPS. Related and unrelated individuals included, using cluster-robust SE. GPS = genome-wide polygenic score. *f* = fraction of causal markers. *b* = unstandardized regression coefficient. Underlined typeset represents most predictive *f* for each subdomain at each age. Bold typeset represents significance under corrected *q* <.05 threshold (FDR-adjusted *P* <.020).

Supplementary Table 18. Multiple Linear Regression Results for Subdomain Mean Scores Regressed on Major Depressive Disorder GPS and Schizophrenia GPS

		MDD GPS				Schizophrenia GPS			
	N	*f*	*b* (SE)	*z (P)*	*ß*	*f*	*b* (SE)	*z (P)*	*ß*
Age 16									
Flat affect	6005	**0.3**	**0.017 (0.005)**	**3.169 (.002)**	**0.044**	1	0.011 (0.005)	2.089 (.037)	0.029
Alogia	6006	1	0.009 (0.008)	1.154 (.249)	0.015	0.3	0.005 (0.008)	0.678 (.498)	0.009
Avolition	5995	**0.3**	**0.031 (0.008)**	**4.075 (<.001)**	**0.055**	0.01	-0.007 (0.008)	-0.898 (.369)	-0.013
Anhedonia	5971	**1**	**0.029 (0.009)**	**3.254 (.001)**	**0.045**	0.3	0.003 (0.009)	0.307 (.758)	0.004
Asociality	5971	**1**	**0.029 (0.007)**	**4.436 (<.001)**	**0.057**	0.01	-0.010 (0.008)	-1.229 (.219)	-0.019
Age 17									
Flat affect	1818	0.3	0.013 (0.012)	1.084 (.278)	0.027	1	0.025 (0.012)	2.009 (.045)	0.052
Alogia	1815	1	0.019 (0.015)	1.225 (.220)	0.029	1	0.003 (0.016)	0.202 (.840)	0.005
Avolition	1816	**1**	**0.049 (0.016)**	**3.036 (.002)**	**0.077**	1	0.030 (0.016)	1.885 (.059)	0.046
Anhedonia	1807	1	0.045 (0.019)	2.327 (.020)	0.062	0.3	0.010 (0.017)	0.578 (.563)	0.014
Asociality	1794	1	0.027 (0.016)	1.651 (.099)	0.043	0.01	-0.021 (0.016)	-1.299 (.194)	-0.033
Age 22									
Flat affect	6274	**0.3**	**0.019 (0.006)**	**3.275 (.001)**	**0.042**	0.01	-0.006 (0.006)	-1.043 (.297)	-0.013
Alogia	6278	1	0.009 (0.008)	1.062 (.288)	0.014	0.01	0.001 (0.008)	0.133 (.894)	0.002
Avolition	6276	**1**	**0.029 (0.008)**	**3.565 (<.001)**	**0.046**	0.01	-0.009 (0.008)	-1.033 (.302)	-0.014
Anhedonia	6251	**1**	**0.032 (0.009)**	**3.424 (.001)**	**0.046**	0.3	-0.012 (0.009)	-1.314 (.189)	-0.018
Asociality	6259	**0.3**	**0.029 (0.008)**	**3.628 (<.001)**	**0.048**	1	0.001 (0.008)	0.179 (.858)	0.002

Note: Subdomains regressed on schizophrenia and MDD GPS jointly for the most predictive GPS *f* (Table 2). Related and unrelated individuals included, using cluster-robust SE. GPS = genome-wide polygenic score. MDD = major depressive disorder. *f* = fraction of causal markers. *b* = unstandardized regression coefficient. *ß* = standardized regression coefficient. Bold typeset represents significance under corrected *q* <.05 threshold (FDR-adjusted *P* <.015).

Supplementary Table 19. Pairwise Wald Test Results for Subdomain Mean Scores Regressed on Major Depressive Disorder GPS and Schizophrenia GPS

	MDD GPS				Schizophrenia GPS			
	Flat affect	Alogia	Avolition	Anhedonia	Flat affect	Alogia	Avolition	Anhedonia
Age 16								
Alogia	1.937 (.164)				1.648 (.199)			
Avolition	2.612 (.106)	**7.141 (.008)**			3.764 (.052)	0.770 (.380)		
Anhedonia	1.721 (.190)	**4.752 (.029)**	0.004 (.950)		0.504 (.478)	0.057 (.811)	0.834 (.361)	
Asociality	1.719 (.190)	**5.189 (.023)**	0.060 (.322)	0.024 (.876)	**4.124 (.042)**	1.492 (.222)	0.116 (.733)	1.818 (.178)
Age 17								
Alogia	0.046 (.830)				2.365 (.124)			
Avolition	**6.231 (.013)**	**4.627 (.031)**			0.550 (.458)	4.708 (.030)		
Anhedonia	2.871 (.090)	1.179 (.181)	0.223 (.637)		0.405 (.524)	0.300 (.583)	1.097 (.295)	
Asociality	0.179 (.672)	0.016 (.900)	3.574 (.059)	2.036 (.154)	**4.420 (.035)**	1.235 (.267)	**5.554 (.018)**	1.450 (.229)
Age 22								
Alogia	1.868 (.172)				0.719 (.396)			
Avolition	1.727 (.189)	**5.356 (.021)**			0.076 (.782)	0.960 (.327)		
Anhedonia	1.913 (.167)	**5.190 (.023)**	0.135 (.714)		0.304 (.582)	0.551 (.458)	0.004 (.950)	
Asociality	2.280 (.131)	**5.287 (.021)**	0.106 (.745)	0.002 (.964)	1.126 (.289)	0.103 (.789)	0.980 (.322)	2.265 (.132)

Note: Subdomains regressed on MDD GPS and schizophrenia GPS separately. Related and unrelated individuals included, using cluster-robust SE. Wald tests conducted for the most predictive GPS *f* (Table 2). Wald tests (*df* = 1) conducted for the difference between the standardized regression coefficients. *P* values (shown in parentheses) indicate significance of difference. GPS = genome-wide polygenic score. MDD = major depressive disorder. *f* = fraction of causal markers. Bold typeset represents significance at *P* <.05.

Supplementary Figure 1. Plot of the First and Second Principal Components of Ancestry

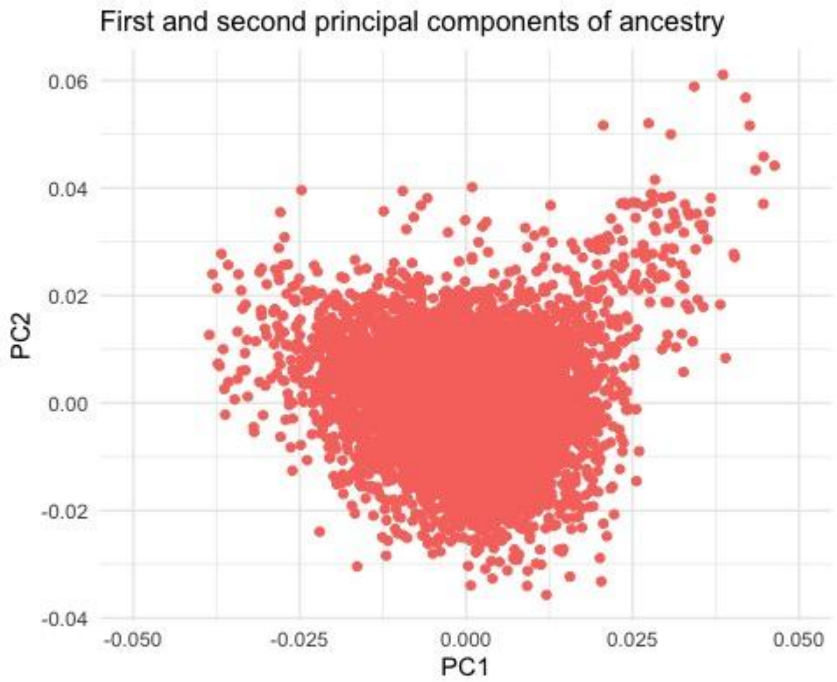

Note. PC = principal component. PCs generated using data from the genotypic sample (see Supplementary Information 2 and 3). Raw GPSs were regressed on the PCs (1-10) prior to analysis.

Supplementary Figure 2. Plots of Subdomain Mean Scores by Major Depressive Disorder GPS Decile Group

A.

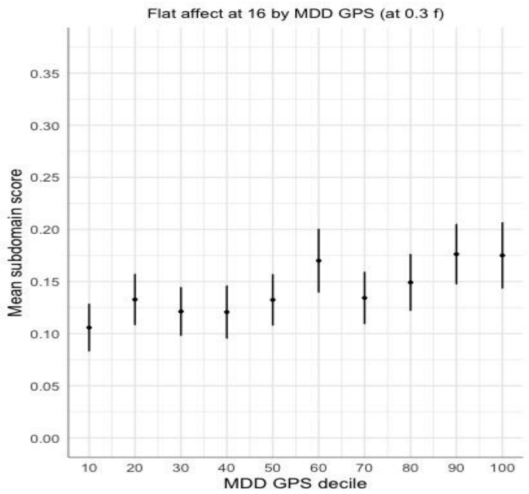

B.

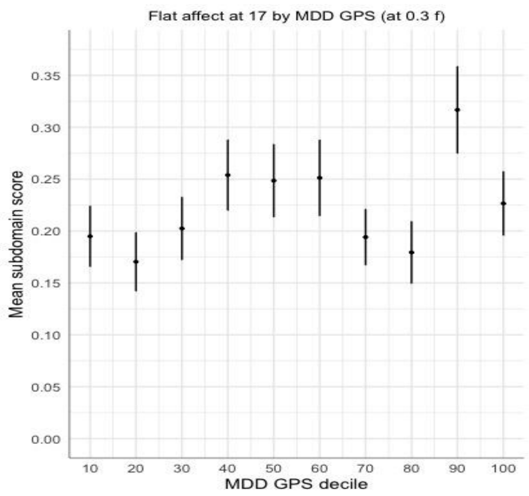

C.

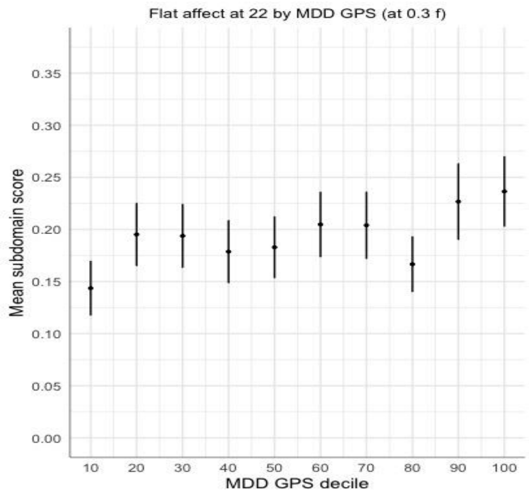

D.

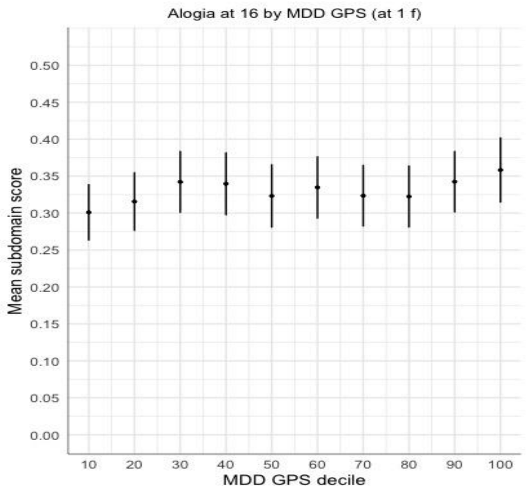

E.

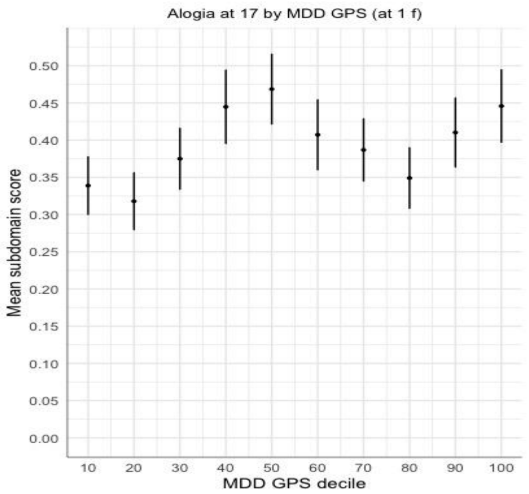

F.

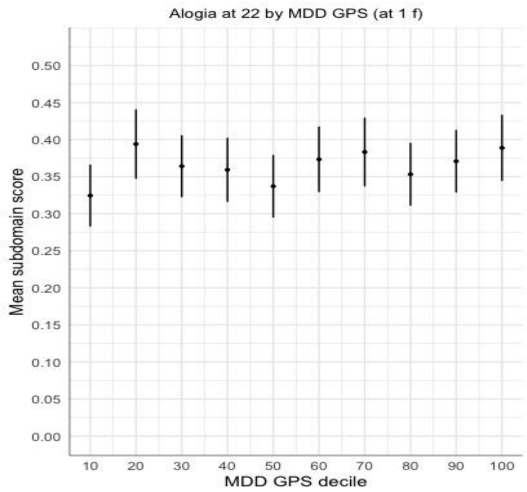

Supplementary Figure 2. Plots of Subdomain Mean Scores by Major Depressive Disorder GPS Decile Group

G.

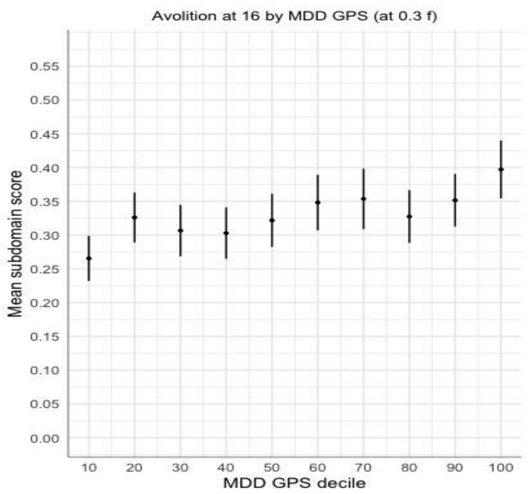

H.

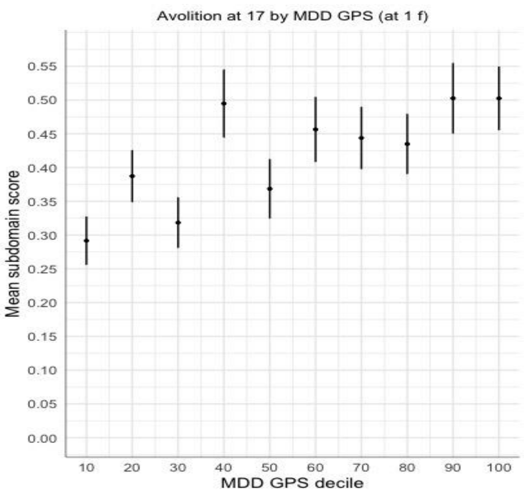

I.

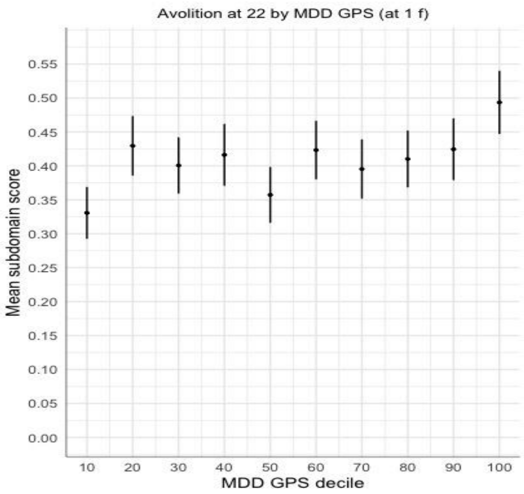

J.

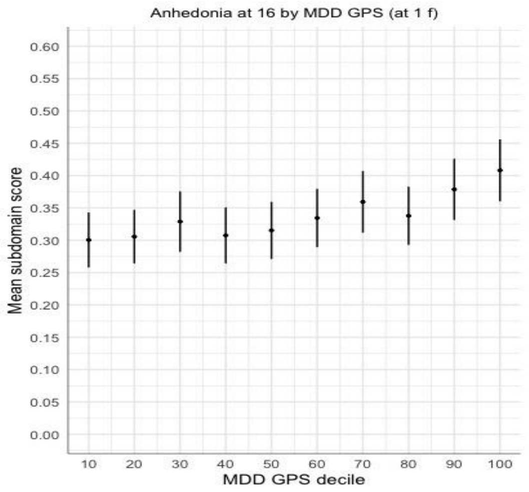

K.

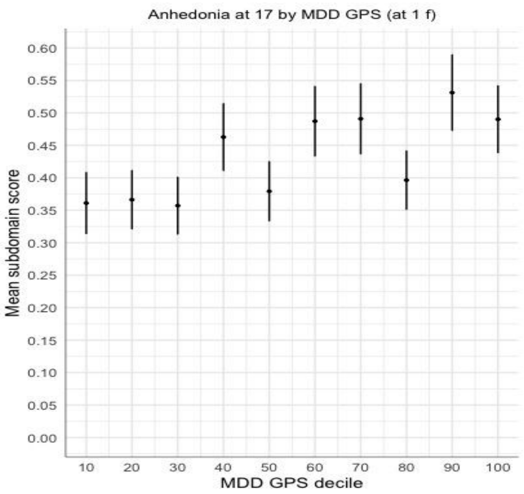

L.

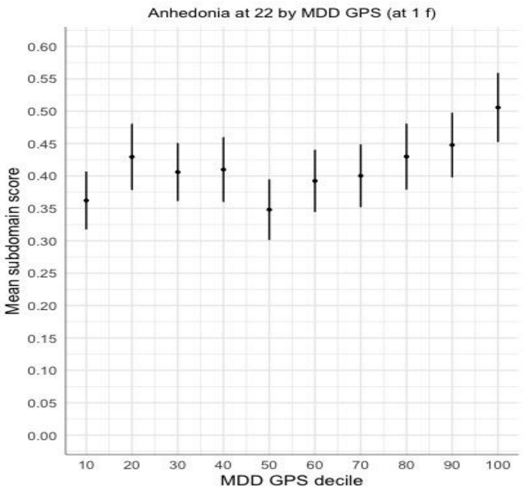

Supplementary Figure 2. Plots of Subdomain Mean Scores by Major Depressive Disorder GPS Decile Group

M.

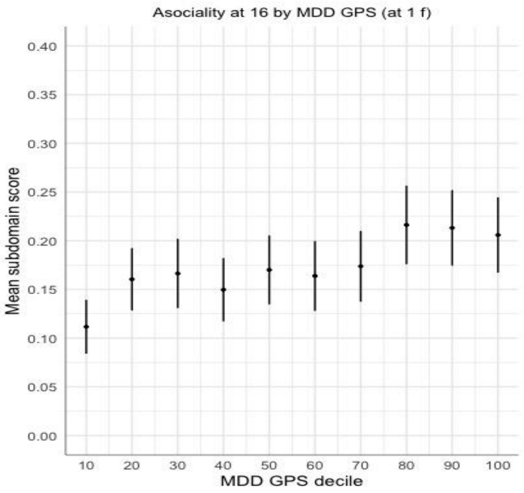

N.

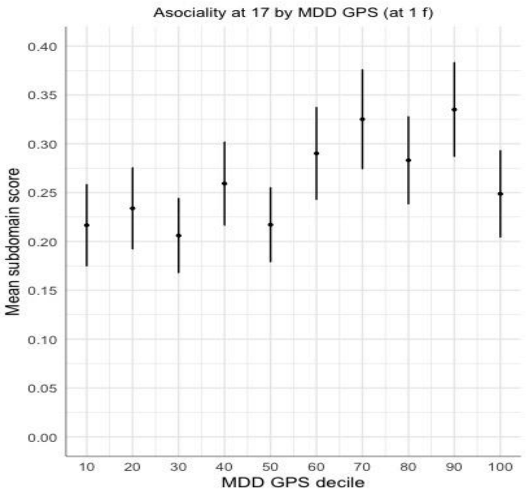

O.

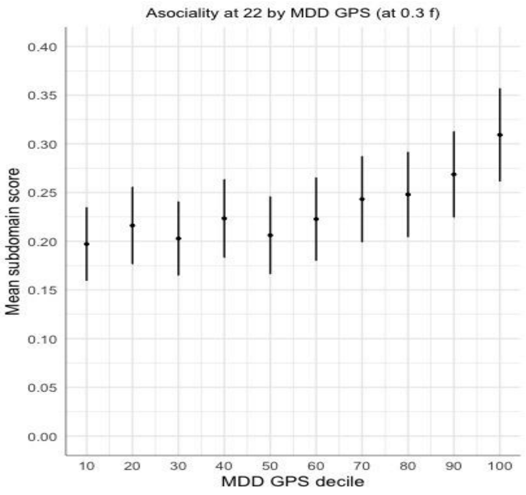

Note. GPS = genome-wide polygenic score. MDD = major depressive disorder. f = fraction of causal markers. Errors bars represent 95% confidence intervals. GPS at each age at the most predictive GPS f (see Table 2).

Supplementary Figure 3. Plots of Subdomain Mean Scores by Schizophrenia GPS Decile Group

A.

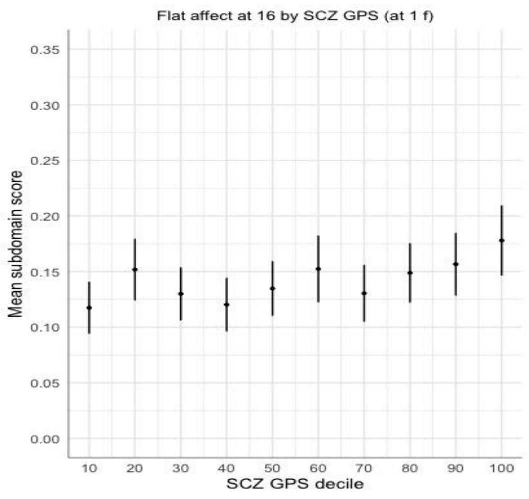

B.

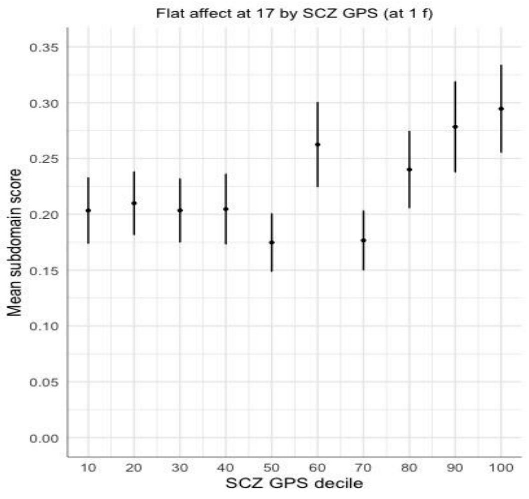

C.

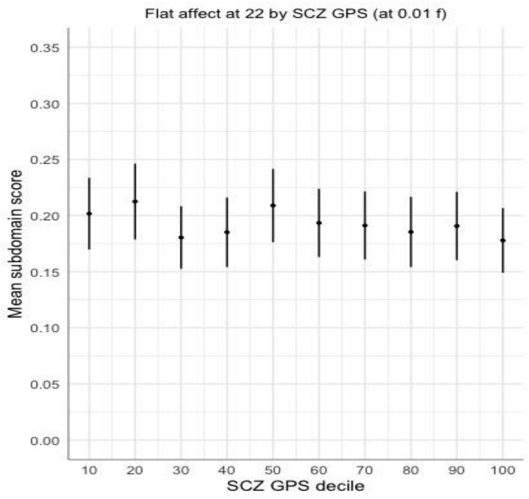

D.

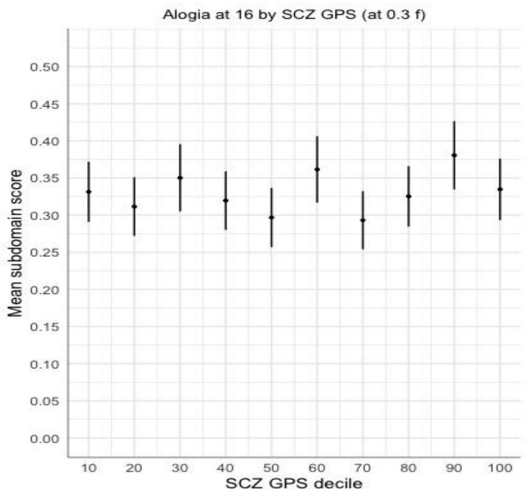

E.

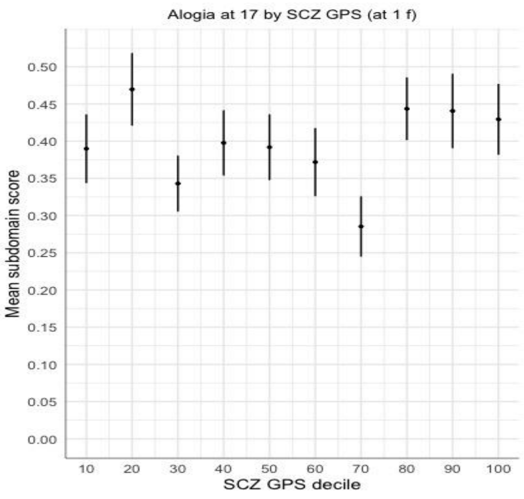

F.

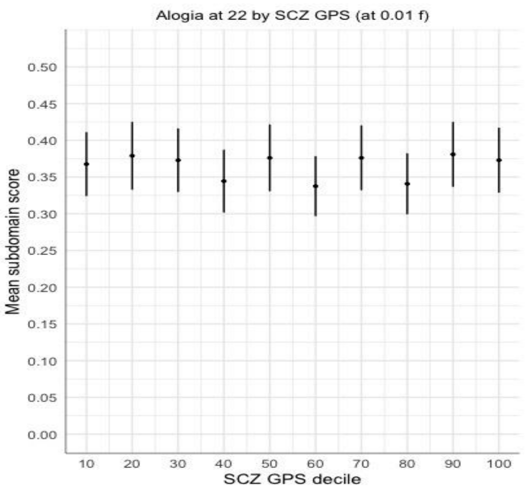

Supplementary Figure 3. Plots of Subdomain Mean Scores by Schizophrenia GPS Decile Group

G.

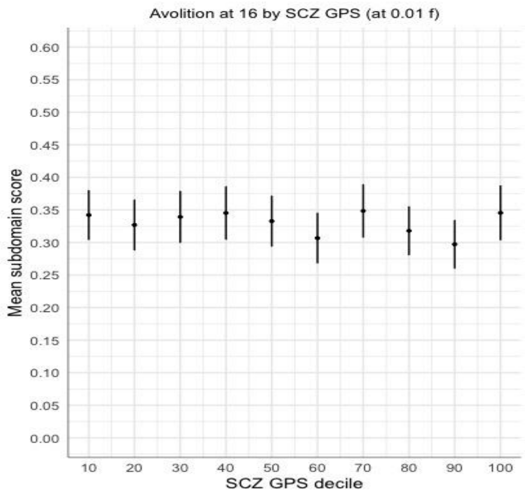

H.

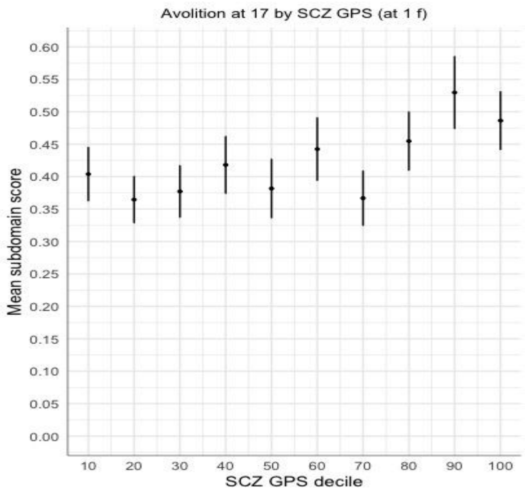

I.

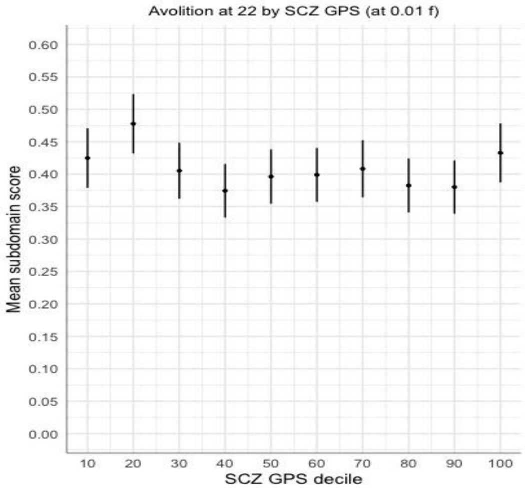

J.

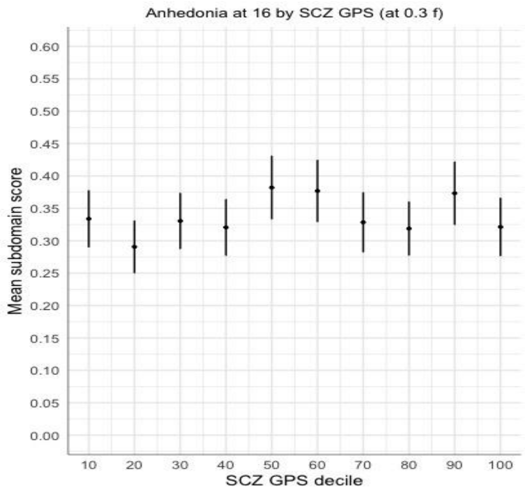

K.

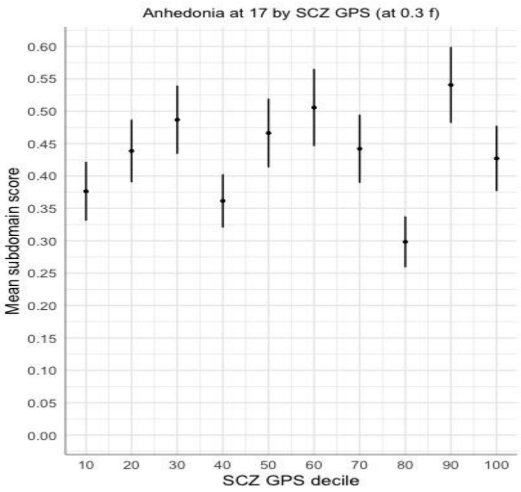

L.

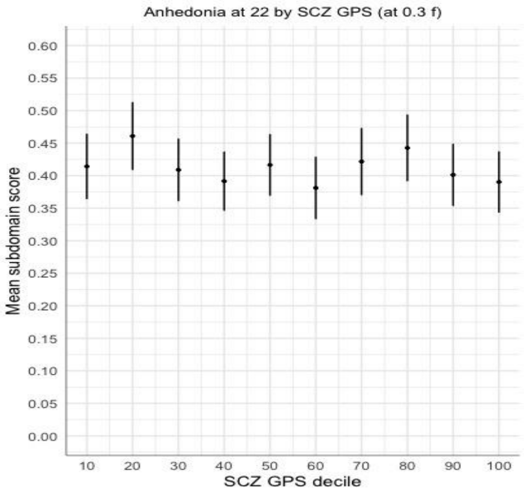

Supplementary Figure 3. Plots of Subdomain Mean Scores by Schizophrenia GPS Decile Group

M.

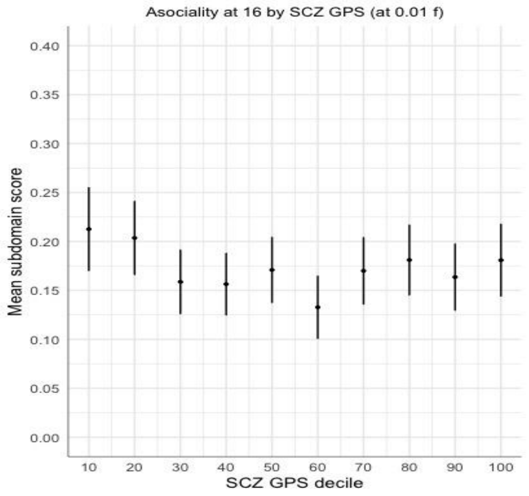

N.

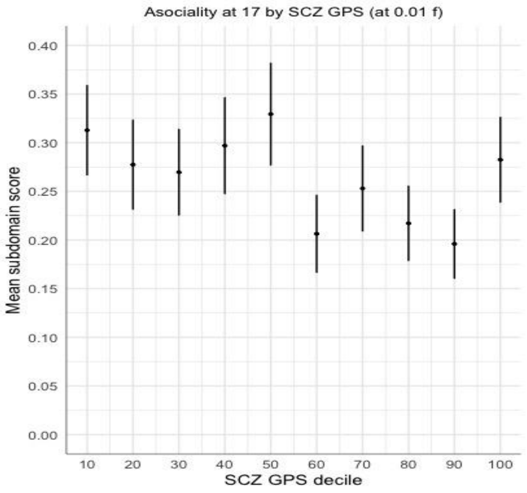

O.

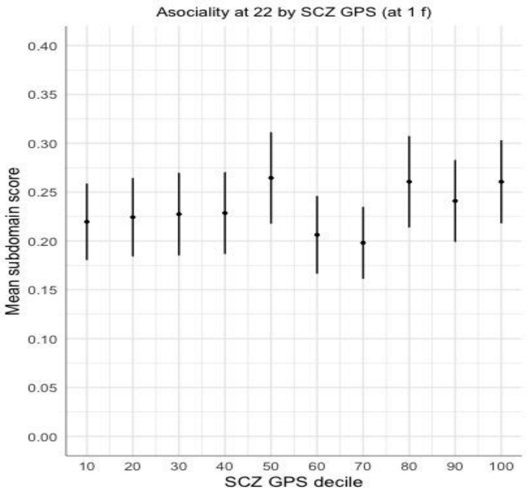

Note. GPS = genome-wide polygenic score. SCZ = schizophrenia. f = fraction of causal markers. Errors bars represent 95% confidence intervals. GPS at each age at the most predictive GPS f (see Table 2).
